# Nuciferine Suppresses Inflammatory Hepatocarcinogenesis by Targeting cPLA2‐Dependent Arachidonic Acid Metabolism

**DOI:** 10.1002/advs.78212

**Published:** 2026-10-08

**Authors:** Jing Sun, Xinyan Wu, Shuwei Luo, Yilang Liu, Jie Liu, Jun Lu, Weimin Wang, Xiao Feng, Tuo Zhang, Dipak Panigrahy, Jianjun Deng, Xiaojun Liao, Haixia Yang

**Affiliations:** ^1^ College of Food Science and Nutritional Engineering China Agricultural University Beijing China; ^2^ Department of General Surgery Dazhou Central Hospital Dazhou China; ^3^ Department of General Surgery Peking University Third Hospital Beijing China; ^4^ Center For Vascular Biology Research Beth Israel Deaconess Medical Center Harvard Medical School Boston Massachusetts USA; ^5^ State Key Laboratory of Vegetable Biobreeding Institute of Vegetables and Flowers Chinese Academy of Agricultural Sciences Beijing China

**Keywords:** arachidonic acid metabolism, cytosolic phospholipase A2, hepatocellular carcinoma, nuciferine

## Abstract

Chronic inflammation is a hallmark of hepatocellular carcinoma (HCC) and is closely linked to aberrant arachidonic acid (AA) metabolism; however, whether AA mobilization is driven by defined upstream regulatory mechanisms and can be therapeutically targeted remains largely unresolved. Here, we identify cytosolic phospholipase A2 (cPLA2) as a central regulator of AA‐dependent inflammatory signaling in hepatocarcinogenesis and a direct pharmacological target of nuciferine (NF). Using diethylnitrosamine (DEN)/carbon tetrachloride (CCl_4_)‐induced HCC mouse models with transcriptomic and lipidomic profiling, we show that cPLA2 activation is progressively elevated during tumorigenesis, leading to excessive eicosanoid production that fuels NF‐κB‐driven inflammation. NF markedly suppresses tumor burden, fibrosis, and inflammatory signaling, accompanied by broad inhibition of AA metabolism. Mechanistically, NF directly binds to the C2 domain of cPLA2, disrupting Ca^2^^+^‐dependent membrane translocation and attenuating its activation. Importantly, *Pla2g4a* deficiency significantly attenuates hepatocarcinogenesis and abolishes the therapeutic effects of NF, establishing cPLA2 as essential for its antitumor activity. In human HCC, increased cPLA2 activation correlates with increased AA metabolism, inflammatory signatures, and poor patient survival. These findings uncover cPLA2‐dependent AA metabolism as a critical inflammatory axis in HCC and identify NF as a direct cPLA2‐targeting agent, highlighting a pharmacologically actionable metabolic node for liver cancer therapy.

## Graphical abstract

Nuciferine directly binds the C2 domain of cPLA2, impairing its Ca^2+^‐dependent membrane translocation and activation. By suppressing arachidonic acid release and downstream pro‐inflammatory eicosanoid production, nuciferine attenuates NF‐κB signaling and inflammatory hepatocarcinogenesis, revealing cPLA2 as a direct molecular target underlying its antitumor activity.

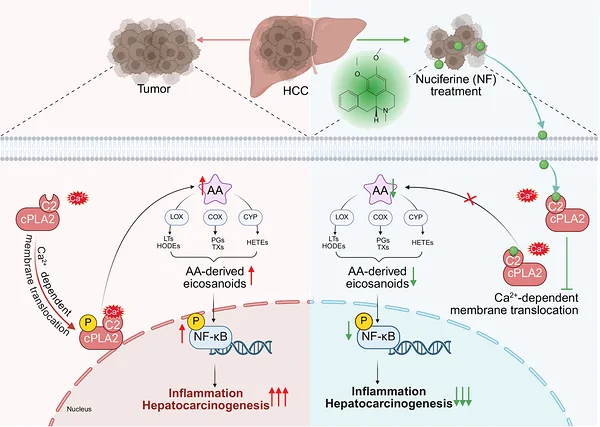

**Abbreviations**
12‐HETE: 12‐hydroxyeicosatetraenoic acid
13‐HODE: 13‐hydroxyoctadecadienoic acid
AA: arachidonic acid
ALT: alanine aminotransferase
AP‐1: activator protein‐1
AST: aspartate aminotransferase
Ccl2: C‐C chemokine ligand 2
CCl_4_
: carbon tetrachloride
CD: Circular dichroism
CETSA: cellular thermal shift assay
CMC‐Na: sodium carboxymethyl cellulose
COX: cyclooxygenase
Cxcl1: chemokine C‐X‐C motif Ligand 1
CYP: cytochrome P450
DARTS: drug affinity responsive target stability
DEN: diethylnitrosamine
DFI: disease free interval
ELISA: enzyme‐linked immunosorbent assay
FRET: fluorescence resonance energy transfer
GSEA: gene set enrichment analysis
HCC: hepatocellular carcinoma
IL‐1β: interleukin‐1β
IL‐6: interleukin‐6
KEGG: kyoto encyclopedia of genes and genomes
KO: knockout
LOX: lipoxygenase
NF: nuciferine
NF‐κB: nuclear transcription factor‐kappa B
OPLS‐DA: orthogonal partial least squares discriminant analysis
OS: overall survival
PBS: phosphate buffered saline
PCNA: proliferating cell nuclear antigen
PFI: progression free interval
PGs: prostaglandins
SPR: surface plasmon resonance
TNF‐α: Tumor necrosis factor α
TXB2: thromboxane B2
VIP: variable importance in projection
WT: wild‐type

## 1 Introduction

Hepatocellular carcinoma (HCC) remains a leading cause of cancer‐related mortality worldwide and most commonly develops in the context of chronic liver inflammation and fibrosis [1, 2]. Despite recent advances in systemic therapies, including immune checkpoint inhibitors and targeted kinase inhibitors, long‐term survival for patients with advanced HCC remains limited [2, 3, 4]. Therefore, a deeper mechanistic understanding of inflammation‐driven hepatocarcinogenesis is essential for identifying therapeutically actionable vulnerabilities.

Chronic hepatic inflammation promotes tumor initiation and progression through sustained production of bioactive lipid mediators, cytokines, and reactive oxygen species [5]. Among these, arachidonic acid (AA)‐derived eicosanoids play a central role in shaping the inflammatory tumor microenvironment [6, 7, 8]. Elevated AA metabolism has been associated with enhanced tumor cell proliferation, angiogenesis, and immune modulation in liver cancer [9, 10, 11]. Although inhibition of downstream enzymes such as cyclooxygenases can reduce eicosanoid production, systemic blockade of these enzymes is limited by gastrointestinal and cardiovascular toxicity [12], highlighting the need to identify upstream regulatory mechanisms that more selectively control inflammatory lipid flux in HCC.

Cytosolic phospholipase A2 (cPLA2, encoded by *PLA2G4A*) catalyzes the rate‐limiting hydrolysis of membrane phospholipids to liberate AA [13, 14]. Activation of cPLA2 requires a two‐step process: Ca^2+^‐dependent membrane translocation mediated by its N‐terminal C2 domain, followed by phosphorylation‐dependent catalytic activation [13, 15, 16]. The C2 domain enables cPLA2 to dock onto perinuclear and endoplasmic reticulum membranes, positioning the enzyme in proximity to phospholipid substrates [17, 18]. This membrane recruitment represents a prerequisite for AA release and subsequent eicosanoid production [19]. cPLA2 is a well‑established key regulator of inflammation [20, 21]. Its activation drives the increased production of prostaglandins in liver cirrhosis [22], counteracts the growth‑inhibitory effects of TGF‑β on hepatocytes [23], and promotes HCC cell growth and invasiveness via the cPLA2/COX‑2/PGE2 pathway [24]. Moreover, S100A10‑mediated activation of the cPLA2/5‑LOX axis reprograms lipid metabolism, elevates LTB4, and promotes CD8^+^ T‑cell exhaustion, facilitating immune evasion in HCC [25]. These observations link cPLA2 to HCC progression, yet direct mechanistic evidence in hepatocarcinogenesis remains limited. In particular, whether C2 domain‐dependent membrane translocation constitutes a rate‐limiting step in inflammatory HCC progression has not been systematically investigated. Furthermore, while downstream eicosanoid synthesis has been extensively targeted pharmacologically [26], it remains unclear whether the membrane recruitment step of cPLA2 is therapeutically druggable in vivo.

Natural compounds have historically provided valuable tools for interrogating disease‐relevant pathways when their molecular targets are clearly defined [27, 28, 29]. Nuciferine (NF), a bioactive aporphine alkaloid isolated from *Nelumbo nucifera*, has been reported to exert anti‐inflammatory and antitumor effects in multiple experimental contexts [30, 31, 32]. However, its precise molecular target and relevance to inflammatory lipid metabolism in HCC have not been elucidated. Whether NF modulates the activation of cPLA2, particularly its C2 domain‐dependent membrane translocation, remains unknown.

In this work, we demonstrate that cPLA2 activation is markedly enhanced during hepatocarcinogenesis in both murine models and HCC patients. Through integrated lipidomic profiling, structural analyses, and biochemical assays, we show that NF directly binds the C2 domain of cPLA2, disrupts Ca^2+^‐dependent membrane docking, and suppresses inflammatory lipid remodeling. Specifically, our study (1) identifies cPLA2 as a direct molecular target of NF; (2) demonstrates that NF binds to the C2 domain of cPLA2 and impairs its Ca^2+^‐dependent membrane translocation, thereby suppressing arachidonic acid metabolism; and (3) provides complementary pharmacological, biochemical, and genetic evidence supporting the functional importance of cPLA2 in mediating the antitumor effects of NF.

## 2 Materials and Methods

Detailed experimental procedures are provided in the supplementary materials and methods.

## 3 Results

### 3.1 NF Treatment Alleviates the Tumor Load in Mice With HCC

To evaluate the therapeutic potential of NF in inflammation‐driven hepatocarcinogenesis, we employed DEN/CCl_4_‐induced HCC models (Figure 1A). Compared with the vehicle‐treated group, NF administration significantly reduced tumor number, tumor size, accompanied by a lower liver/body weight ratio (Figure 1B,C). Histological analysis revealed decreased necrotic areas in NF‐treated livers (Figure 1D). Consistently, NF intervention lowered aspartate aminotransferase (AST) levels, with plasma alanine aminotransferase (ALT) also exhibiting a decreasing trend (Figure 1E), indicating amelioration of liver injury. Fibrotic remodeling was also attenuated, as evidenced by reduced Sirius Red staining and decreased expression of the fibrosis‑related gene *Ctgf*, with *Col1a1* and *Timp1* showing a decreasing trend in the NF group relative to the Model group (Figure 1F,G). Moreover, immunohistochemical analysis demonstrated reduced hepatocyte proliferation in NF‐treated mice, reflected by lower Ki67 and proliferating cell nuclear antigen (PCNA) staining (Figure 1H). Altogether, these findings indicate that NF mitigates tumor burden, liver injury, fibrosis, and proliferative activity in experimental HCC.

**FIGURE 1:**
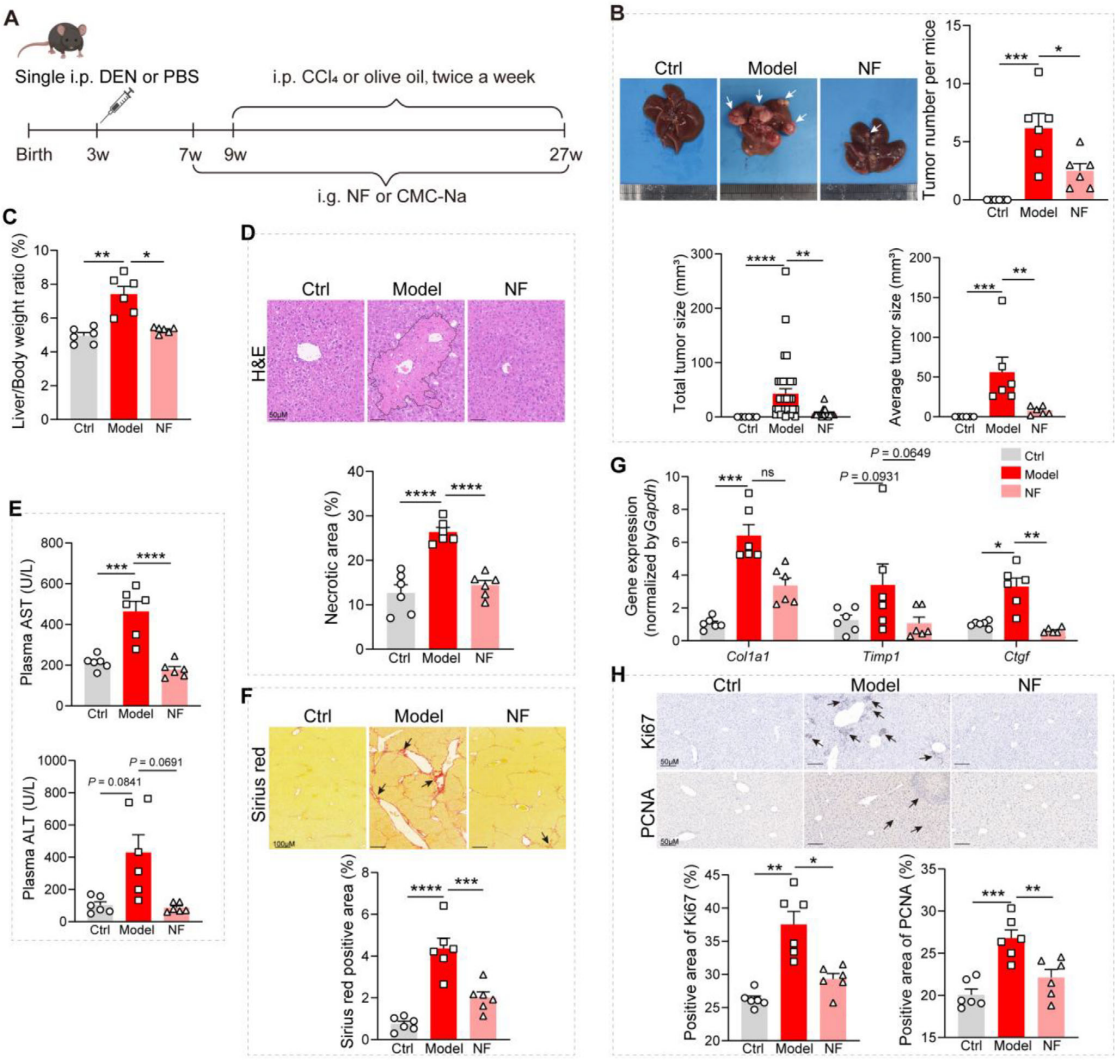
NF suppresses DEN/CCl_4_‐induced HCC tumorigenesis in vivo. (A) Scheme of animal experiment (*n*  =  6). (B) Quantification of liver tumors in mice (*n*  =  6). The scatter plot of total tumor size (mm^3^) represents individual tumor nodules, whereas the animal sample size is n = 6 mice per group. (C) Liver/body weight ratios in mice (*n*  =  6). (D) Representative images of H&E‐stained liver sections and quantification of necrotic area (*n* = 6). Scale bar: 50 µm. (E) Plasma AST and ALT levels in mice (*n* = 6). (F) Representative images of Sirius Red‐stained liver sections and quantification of positive area (*n*  =  6). Scale bar: 100 µm. (G) mRNA levels of hepatic fibrosis markers (*Col1a1*, *Timp1*, *Ctgf*) were quantified by RT‐qPCR (*n* = 6). (H) Representative images and quantification of Ki67‐ and PCNA‐positive areas in liver sections (*n* = 6). Scale bar: 50 µm. Data are presented as mean ± SEM. Statistical analyses were performed by one‐way ANOVA. ^∗^ *p* <0.05, ^∗∗^ *p* <0.01, ^∗∗∗^ *p* <0.001, ^∗∗∗∗^ *p* <0.0001, ns, not significant.

### 3.2 NF Suppresses Hepatic AA Metabolism in Mice With HCC

To investigate the mechanisms underlying NF‐mediated suppression of HCC progression, we performed transcriptomics combined with targeted lipidomics (Figure 2A). Comparative analysis using Venn diagrams revealed distinct transcriptional profiles among control, HCC model, and NF‐treated groups (Figure 2B). Subsequent KEGG annotation of the overlapping differentially expressed genes demonstrated significant enrichment of lipid metabolism pathways (Figure 2C,D). Notably, NF treatment partially reversed these transcriptional alterations, particularly genes associated with inflammatory lipid signaling (Figure 2C,D). KEGG pathway enrichment analysis of lipid‐related differentially expressed genes identified AA metabolism as one of the most significantly dysregulated pathways and NF treatment markedly attenuated the activation of this pathway (Figure 2E), suggesting that modulation of AA metabolism may contribute to its therapeutic effect.

**FIGURE 2:**
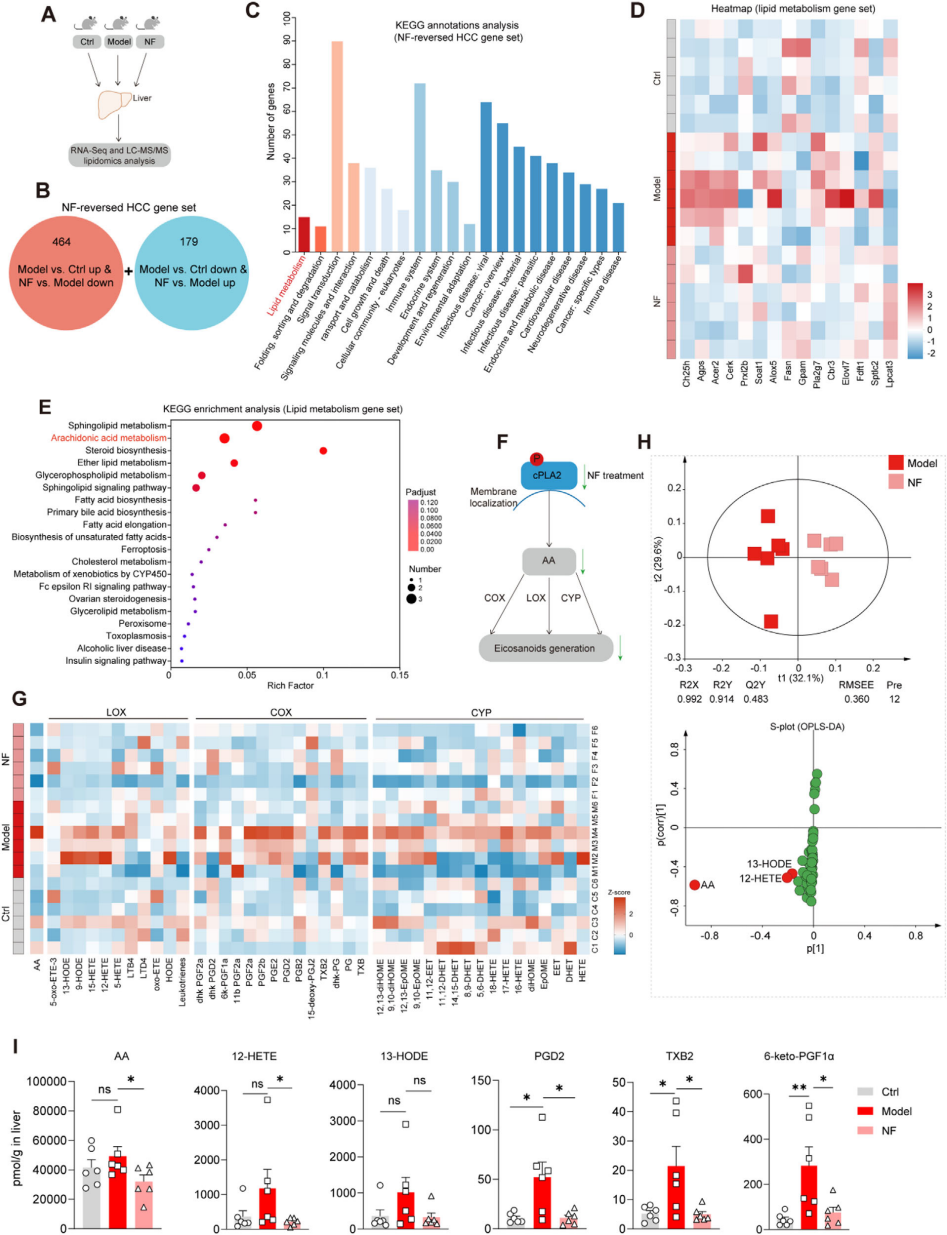
NF suppresses hepatic AA metabolism during HCC progression. (A) Schematic overview of transcriptomic and LC‐MS/MS‐based lipidomic analyses. (B) Venn diagram depicting the NF‑reversed HCC gene set, defined as the union of two directional sets: (i) genes upregulated in Model vs. Ctrl and downregulated in NF vs. Model (padj < 0.05, FC > 1.5), and (ii) genes downregulated in Model vs. Ctrl and upregulated in NF vs. Model under the same thresholds (*n* = 6). The total reversed gene set comprises 643 genes (464 from condition i, 179 from condition ii). (C) KEGG pathway classification of the NF‑reversed HCC gene set, highlighting lipid‑metabolism‑related genes. (D) Heatmap showing expression profiles of the 15 lipid‑metabolism‑related genes identified from the reversed gene set in Ctrl, Model, and NF‑treated mice (*n* = 6). (E) KEGG enrichment analysis of the 15 lipid‑metabolism‑related genes, revealing significant enrichment of the arachidonic acid (AA) metabolism pathway. (F) Workflow for analysis of AA and downstream eicosanoids. (G) Heatmap of hepatic AA and LOX‐, COX‐, and CYP‐derived eicosanoids across experimental groups (*n* = 6). (H) OPLS‐DA score plot distinguishing eicosanoid profiles between Model and NF‐treated mice (*n* = 6). The S‐plot identifies metabolites contributing to group separation (VIP > 1). (I) Hepatic levels of AA and AA‑derived eicosanoids downregulated by NF treatment (*n* = 6). Data are presented as mean ± SEM. Statistical analyses were performed using one‐way ANOVA. I: One‑way ANOVA was used for group comparisons, with false‑discovery‑rate (FDR) correction applied using the Benjamini‐Hochberg method for multiple comparisons. ^∗^ *p* <0.05, ^∗∗^ *p* <0.01, ns, not significant.

To further assess changes at the metabolite level, we performed LC‐MS/MS‐based lipidomic profiling of hepatic eicosanoids (Figure 2F), which yielded a total of 43 eicosanoids (µmol/g in liver) (Table S3). Consistent with transcriptomic findings, HCC livers exhibited increased accumulation of AA and multiple downstream LOX‐, COX‐, and cytochrome P450 (CYP)‐derived eicosanoids (Figure 2G). NF treatment significantly reduced hepatic AA levels and broadly decreased the abundance of downstream eicosanoids. Orthogonal partial least squares discriminant analysis (OPLS‐DA) clearly separated Model and NF‐treated groups (Figure 2H), indicating distinct lipidomic signatures. Metabolites with variable importance in projection (VIP) scores >1 included AA, 12‐hydroxyeicosatetraenoic acid (12‐HETE), and 13‐hydroxyoctadecadienoic acid (13‐HODE), suggesting that these lipids contributed prominently to group discrimination. Pro‐inflammatory and pro‐angiogenic mediators, including AA, 12‐HETE, 13‐HODE, thromboxane B2 (TXB2) and 6‑keto‑prostaglandin F1α (6‐keto‐PGF1α), were elevated in HCC livers compared with controls. NF administration reduced the levels of AA and several of these downstream mediators relative to untreated tumor‐bearing mice (Figure 2I). These data indicate that NF suppresses hepatic AA metabolism and attenuates inflammatory eicosanoid production in mice with HCC.

### 3.3 NF Directly Interacts With cPLA2 and Inhibits Its Activity

Given the central role of cPLA2 (encoded by *Pla2g4a*) in releasing AA for eicosanoid biosynthesis, we next investigated whether NF suppresses hepatic AA metabolism by inhibiting cPLA2 expression or activity. RNA‑seq and RT‑qPCR analyses showed that *Pla2g4a* mRNA levels did not differ significantly among the three groups (Figure S1A). In contrast, cPLA2 enzymatic activity was increased in HCC liver tissue and plasma and was significantly reduced following NF administration (Figure 3A). Consistently, western blot and immunofluorescence analyses showed enhanced phosphorylation of cPLA2 in tumor‐bearing mice, which was attenuated by NF (Figure 3B,C). Importantly, because our study focuses on cPLA2 activation, reflected by its phosphorylation and membrane translocation, rather than total protein abundance, the total cPLA2 protein levels shown in Figure 3B do not necessarily parallel the mRNA expression pattern. To determine whether NF directly interacts with cPLA2, we performed the drug affinity responsive target stability (DARTS) assay. NF bound to and stabilized cPLA2 against proteolytic degradation in a concentration‐dependent manner, with the maximal protection observed at 30 µM (Figure 3D). Similarly, cellular thermal shift assays (CETSA) across a temperature gradient (40°C–64°C) showed increased thermal stability of cPLA2 in the presence of NF, consistent with ligand binding in both RAW 264.7 (Figure 3E) and HepG2 cells (Figure S1B). Our mechanistic conclusions are therefore drawn from cPLA2 activation status (phosphorylation and enzymatic activity), rather than its transcriptional upregulation or total protein levels.

**FIGURE 3:**
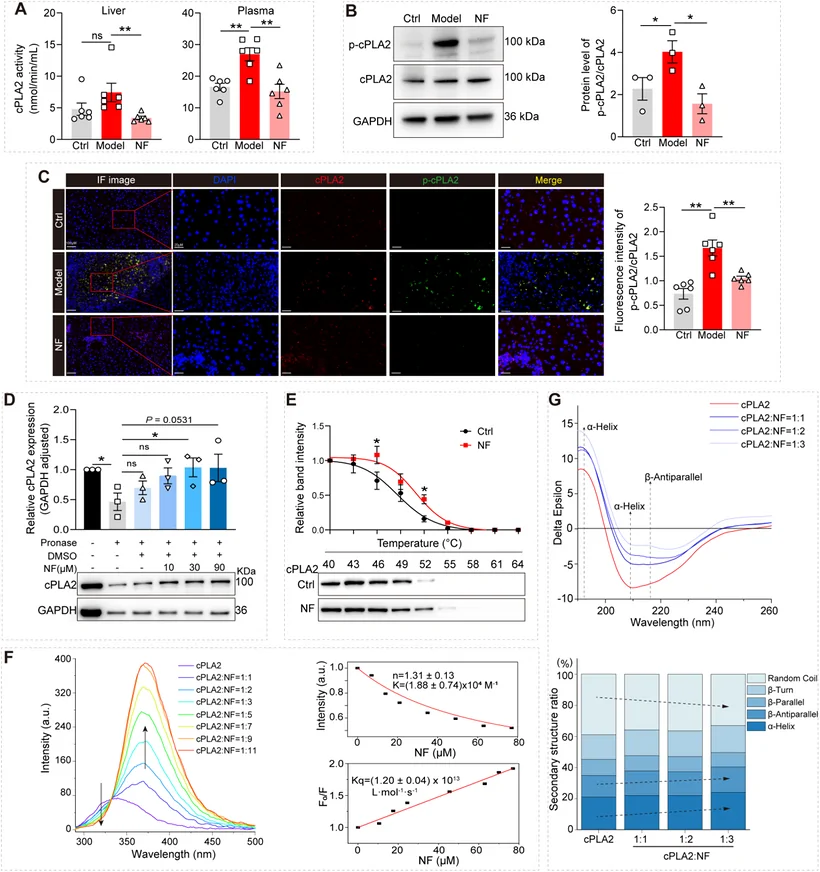
NF directly interacts with cPLA2 and inhibits its activity. (A) cPLA2 enzymatic activity in liver tissue and plasma (*n* = 6). (B) Western blot analysis of phosphorylated and total cPLA2 in liver tissues (*n* = 3). Quantification of phospho‑cPLA2 (Ser505) was performed as the ratio of phospho‑cPLA2 to total cPLA2, with both signals first normalized to GAPDH. (C) IF staining of total cPLA2 (red) and phospho‐cPLA2 (green) in liver sections. (*n* = 6). Scale bars, 100/20 µm. (D) DARTS assay showing pronase resistance of cPLA2 in RAW264.7 cells treated with increasing concentrations of NF. (E) CETSA assessing the thermal stabilization of cPLA2 by NF in RAW264.7 cell lysates (40°C–64°C). GAPDH was used as a loading control. (F) Fluorescence quenching spectra of purified cPLA2 in the presence of NF. Binding constant (K), stoichiometry (n), and Stern‐Volmer constant (Kq) are indicated. (G) CD spectra of cPLA2 with increasing molar ratios of NF. Data in B, D, E are presented as mean ± SEM and representative of 3 independent biological replicates. Statistical analyses were performed using one‐way ANOVA (A‐D) or Student's t‑test (E). ^∗^ *p* <0.05, ^∗∗^ *p* <0.01, ns, not significant.

To further characterize this interaction, recombinant human cPLA2 was expressed and purified, and its enzymatic activity was confirmed (Figure S2A–C). Fluorescence titration assays revealed a concentration‐dependent decrease in intrinsic cPLA2 emission upon NF addition (Figure 3F and Figure S2D). The calculated Stern‐Volmer quenching constant exceeded the diffusion‐controlled limit for biomolecular collisions, supporting a static quenching mechanism consistent with complex formation. Binding analysis yielded a moderate association constant and a stoichiometry approximating 1:1. Spectral overlap between cPLA2 emission and NF absorption, together with the emergence of a new emission peak, suggested fluorescence resonance energy transfer (FRET), further supporting direct molecular interaction (Figure S2E and Figure 3F). Synchronous fluorescence spectroscopy demonstrated that tryptophan (Trp) residues exhibited a red shift indicative of increased solvent exposure, whereas tyrosine (Tyr) residues showed fluorescence quenching without peak displacement (Figure S2F,G), suggesting selective perturbation of the Trp microenvironment upon NF binding. CD analysis further revealed modest but reproducible conformational changes in cPLA2 in the presence of NF, including slight increases in α‐helix and β‐sheet content accompanied by a reduction in random coil structure (Figure 3G and Figure S2H). These data indicate that NF directly binds to cPLA2 and suppresses its enzymatic activation, providing a mechanistic basis for the attenuation of AA metabolism observed in vivo.

### 3.4 NF Binds the C2 Domain of cPLA2 and Impairs Ca^2+^‐Dependent Membrane Translocation

To localize the binding site, molecular docking analysis predicted preferential interaction of NF within the C2 domain of cPLA2, particularly near Ca^2+^‐binding loop regions (Figure 4A). To experimentally validate this prediction, the isolated C2 domain was purified (Figure S3A–C) and subjected to SPR analysis. SPR confirmed direct binding of NF to the C2 domain, yielding a dissociation constant (Kd) of 14.62 µM (Figure 4B), consistent with moderate affinity. Although binding data from the refolded full‑length cPLA2 should be interpreted with caution, the conclusions are supported by the independent SPR results obtained with the isolated C2 domain.

**FIGURE 4:**
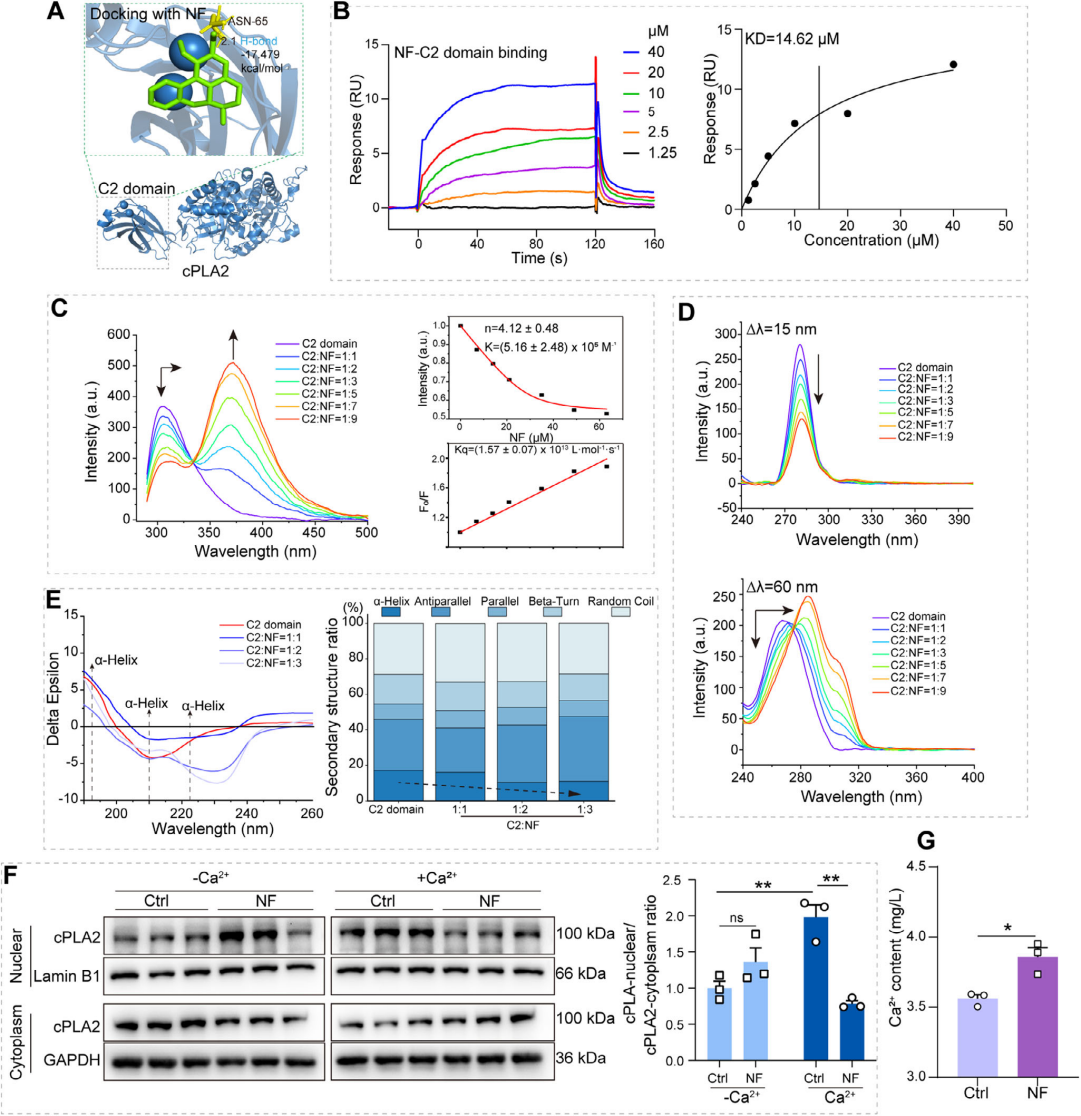
NF binds the C2 domain of cPLA2 and disrupts Ca^2+^‐dependent membrane translocation. (A) Molecular docking models of NF with cPLA2 (PDB ID: 1CJY). (B) SPR analysis of NF binding to the purified C2 domain. (C) Fluorescence‐quenching spectra of the C2 domain in the presence of NF. Binding parameters are shown in the inset. (D) Synchronous fluorescence spectroscopy (△λ = 15 and 60 nm) of C2 domain with increasing NF concentrations. (E) CD spectra of C2 domain in the absence and presence of NF. (F) Subcellular localization of cPLA2 in RAW264.7 cells following Ca^2+^ stimulation with or without NF treatment. Cytosolic and nuclear fractions were analyzed by western blotting. GAPDH and Lamin B1 served as cytosolic and nuclear markers, respectively. (G) ICP‐MS‐based quantitative analysis of free Ca^2+^ released from the cPLA2 C2 domain in the presence or absence of NF (100 µM). Data in (F, G) are presented as mean ± SEM and representative of 3 independent biological replicates. Statistical analyses were performed using Student's t‐test. ^∗^ *p* <0.05, ^∗∗^ *p* <0.01.

Fluorescence titration assay further confirmed NF binding to the C2 domain, as evidenced by concentration‐dependent quenching of intrinsic C2 fluorescence together with the emergence of a new emission peak at 375 nm (Figure 4C and Figure S3D). Spectral overlap between C2 domain emission and NF absorption suggested FRET (Figure S3E). The calculated Stern‐Volmer constant exceeded the diffusion‐controlled limit, supporting a static quenching mechanism indicative of complex formation. Notably, the apparent binding affinity of NF for the isolated C2 domain was higher than that observed for full‐length cPLA2, suggesting increased accessibility of the binding interface in the truncated construct (Figure 4C). Synchronous fluorescence spectroscopy revealed a red‐shift for Trp emission (Δλ = 60 nm), whereas tyr residues exhibited fluorescence quenching without peak displacement (Figure 4D and Figure S3F). Given that the C2 domain contains a single Trp residue (Trp71), these findings suggest that NF binding perturbs the local microenvironment surrounding this residue. CD analysis showed a reduction in α‐helical content within the C2 domain upon NF binding (Figure 4E). Because α‐helical elements contribute to the structural integrity of Ca^2+^‐binding loop regions [13, 33], these conformational alterations may influence membrane interaction dynamics.

Given the established role of the C2 domain in Ca^2+^‐dependent membrane recruitment, we next examined cPLA2 subcellular localization. In RAW264.7 and HepG2 cells, NF did not alter basal cPLA2 distribution under unstimulated conditions (Figure 4F and Figure S3G). However, upon Ca^2+^ stimulation, NF significantly attenuated perinuclear translocation of cPLA2. To assess whether NF competitively disrupts Ca^2+^ binding with C2 domain protein, we conducted an NF intervention experiment using a C2 domain protein‐Ca^2+^ incubation system. The purified C2 domain (20 µM) was preincubated with Ca^2+^ and subsequently treated with or without NF (0 or 100 µM) (Figure 4G). Treatment with NF increased the free Ca^2+^ concentration from 3.56 ± 0.04 mg/L (88.81 ± 1.00 µM) to 3.86 ± 0.09 mg/L (96.29 ± 2.28 µM), corresponding to a net release of 7.5 µM Ca^2+^. Molecular docking predicts Asn65 as a potential binding residue, a key Ca^2+^ coordinating residue within binding site I [34]. These findings suggest that NF primarily interferes with Ca^2+^ coordination at Site I, whereas the Ca^2+^‐binding environment of Site II, Asn95, is not directly disrupted. This may explain why NF does not simultaneously displace both Ca^2+^ ions bound to the C2 domain. In summary, these findings demonstrate that NF competitively disrupts Ca^2+^ binding with the C2 domain protein, thereby preventing Ca^2+^‐dependent membrane translocation and subsequent enzymatic activation during HCC progression.

### 3.5 cPLA2 Deficiency Abolishes the Antitumor Effect of NF

To determine whether cPLA2 is required for the therapeutic efficacy of NF in vivo, we employed cPLA2 knockout (KO) mice (Figure S4A–C) subjected to DEN/CCl_4_‐induced hepatocarcinogenesis (Figure 5A). Two‐way ANOVA analysis revealed an interaction between genotype (wild‐type (WT) vs. cPLA2 KO) and treatment (NF vs. vehicle) on average tumor size, liver injury, liver fibrosis, and abnormal hepatocyte proliferation. In WT mice, NF treatment significantly reduced tumor burden, as evidenced by decreased tumor number and size (Figure 5B), a lower liver/body weight ratio (Figure 5C), improved histological damage (Figure 5D), and reduced plasma ALT and AST levels (Figure 5E). NF treatment also reduced liver fibrosis (Figure 5F), downregulated the expression of fibrosis‐related genes (*Col1a1*, *Ctgf*, and *Timp1*) (Figure 5G), and suppressed hepatocyte proliferation (Figure 5H). In contrast, these therapeutic benefits were abolished in cPLA2 KO mice, with no significant differences observed between NF‐treated and untreated groups, supporting that the anti‐HCC efficacy of NF in vivo is cPLA2‐dependent.

**FIGURE 5:**
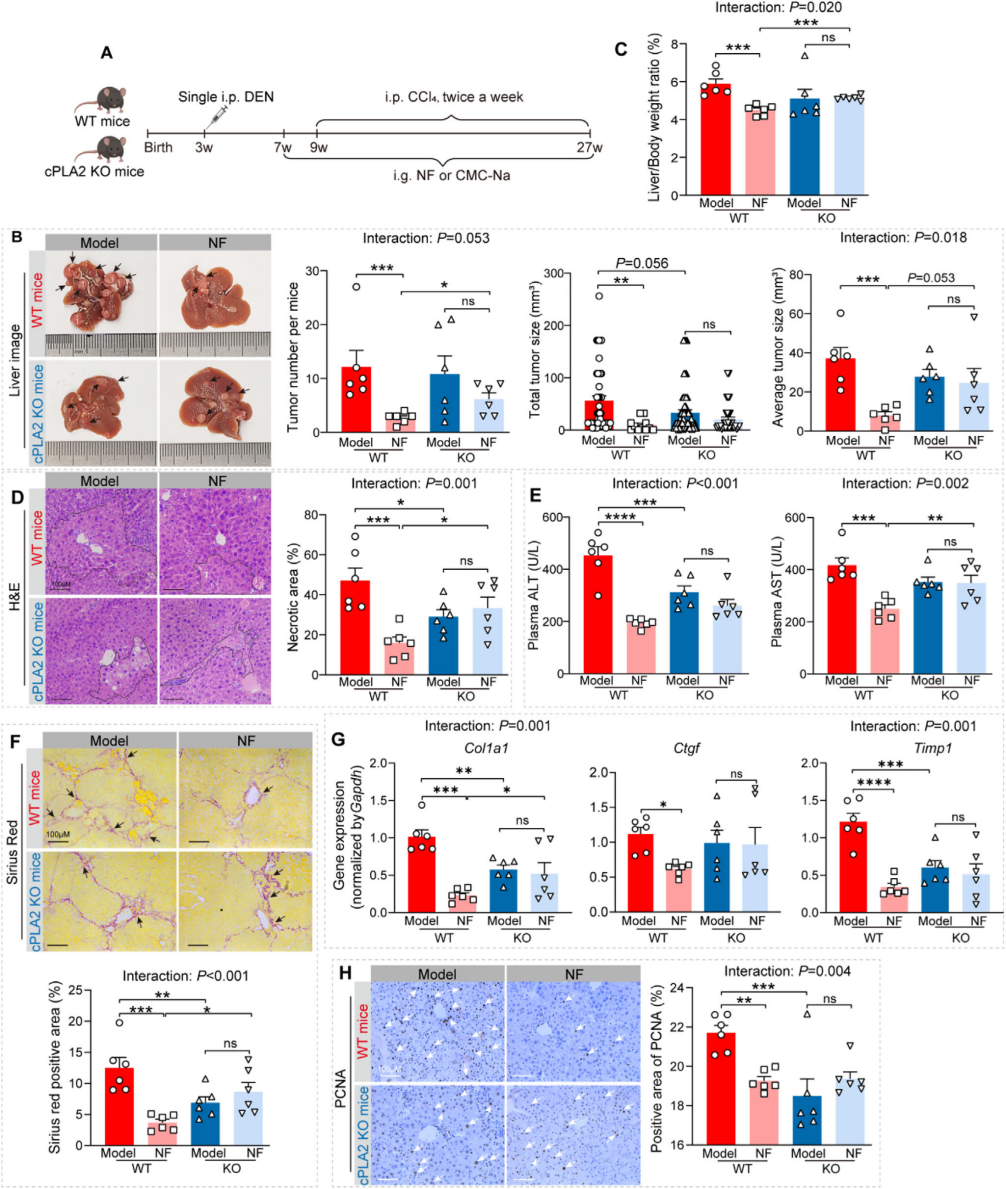
Genetic ablation of cPLA2 abolishes the antitumor effect of NF. (A) Experimental design of DEN/CCl_4_‐induced hepatocarcinogenesis in WT and cPLA2 KO mice with or without NF treatment (*n* = 6). (B) Quantification of liver tumor number and size in mice (*n*  =  6). (C) Liver/body weight ratios in mice (*n*  =  6). (D) Representative H&E staining and quantification of necrotic area. Scale bar: 50 µm. (E) Plasma AST and ALT levels in mice (*n* = 6). (F) Sirius Red staining and quantification of fibrotic area. Scale bar: 100 µm. (G) Hepatic mRNA levels of fibrosis‐associated genes (*n* = 6). (H) PCNA immunohistochemistry and quantification of positive staining. (*n* = 6). Scale bar: 50 µm. Data are presented as mean ± SEM and representative of 6 independent biological replicates. The statistical analysis was performed by two‐way ANOVA. ^∗^ *p* <0.05, ^∗∗^ *p* <0.01, ^∗∗∗^ *p* <0.001, ^∗∗∗∗^ *p* <0.0001, ns, not significant.

### 3.6 NF Suppresses NF‐κB‐Mediated Inflammatory Signaling Through cPLA2 Activation

To investigate downstream inflammatory signaling affected by NF, RNA‐seq followed by gene set enrichment analysis (GSEA) was performed. GSEA confirmed that tumor necrosis factor α (TNF‐α)/nuclear transcription factor‐kappa B (NF‐κB) signaling was significantly upregulated in HCC mice (Figure 6A and Figure S5A). Further analysis showed that NF treatment significantly downregulated this pathway compared with untreated HCC mice (Figure 6A and Figure S5B). Consistently, NF treatment decreased phosphorylation of NF‑κB p65 in liver tissue (Figure 6B) and reduced plasma levels of the pro‑inflammatory cytokines TNF‑α, interleukin‐1β (IL‑1β), and interleukin‐6 (IL‑6) (Figure 6C). Hepatic mRNA expression of inflammatory mediators, including *Il‑1β, Tnf‑α*, activator protein‐1*(Ap‑1)*, chemokine C‐X‐C motif Ligand 1 *(Cxcl1)*, and C‐C chemokine ligand 2 (*Ccl2*), was also downregulated (Figure 6D).

**FIGURE 6:**
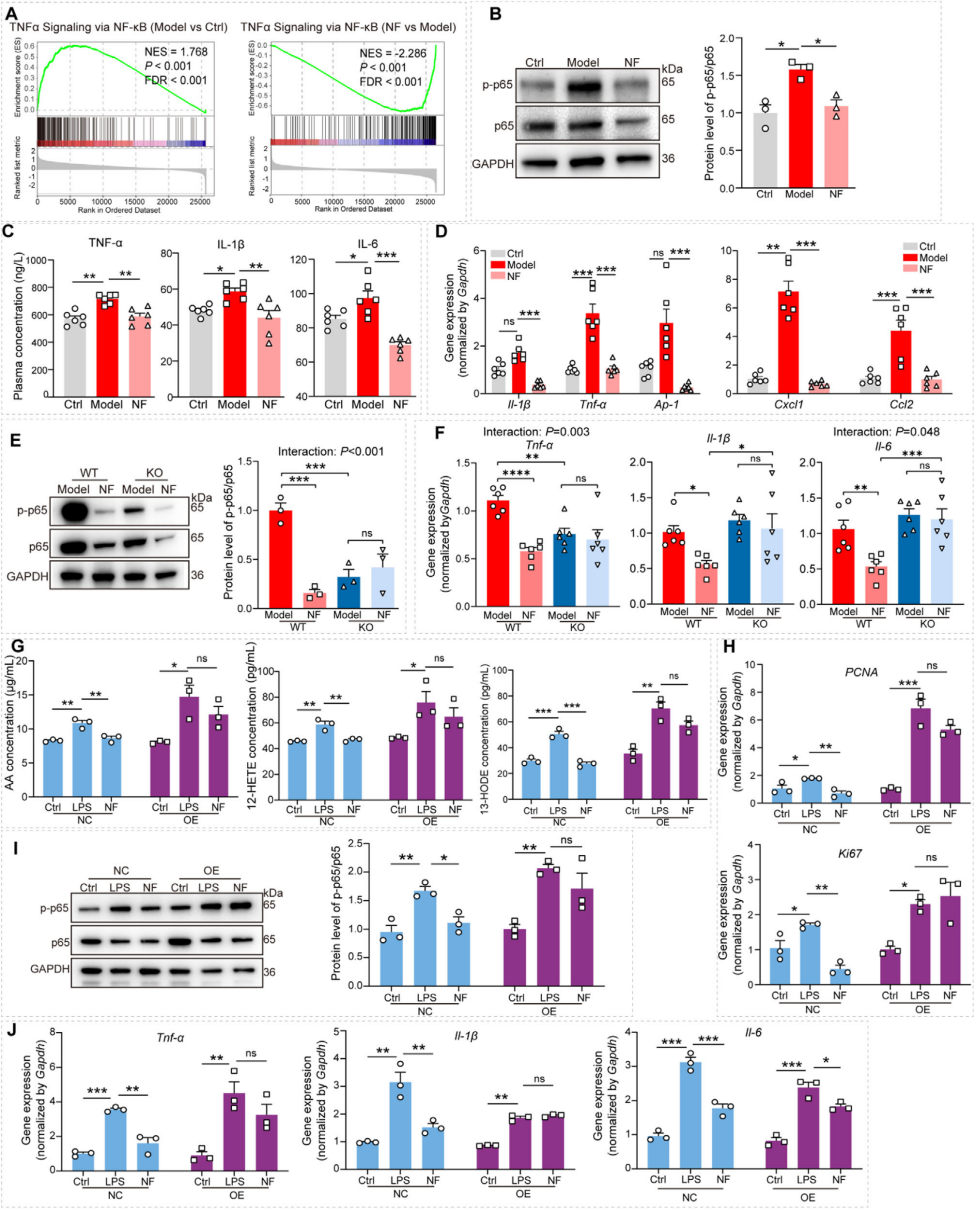
Suppression of NF‐κB signaling by NF is dependent on cPLA2 inhibition. (A) Enrichment score plot for the TNFα signaling via NF‑κB pathway between Model vs Ctrl and NF vs Model groups (*n* = 6). (B) Western blot analysis of NF‐κB p65 phosphorylation in liver tissues (n = 3). (C) Plasma levels of TNF‐α, IL‐1β, and IL‐6 measured by ELISA (*n* = 6). (D) Hepatic mRNA levels of pro‐inflammatory genes (*n* = 6). (E) Western blot analysis of NF‐κB p65 phosphorylation in liver tissues from WT and cPLA2‐KO mice with or without NF treatment (*n* = 3). (F) Hepatic pro‐inflammatory gene expression in WT and cPLA2‐KO mice (*n* = 6). (G) Concentrations of AA, 12‐HETE and 13‐HODE in control and cPLA2‐overexpressing HepG2 cells (*n* = 3). (H) mRNA levels of *PCNA* and *Ki67* in control and cPLA2‑overexpressing HepG2 cells (*n* = 3). (I) Phosphorylation of p65 in control and cPLA2‐overexpressing HepG2 cells (*n* = 3). (J) Inflammatory gene expression in control and cPLA2‐overexpressing HepG2 cells (*n* = 3). Data in (B, E, G‐J) are presented as mean ± SEM and representative of 3 independent biological replicates. Statistical analyses were performed using one‐way ANOVA (B‐D, G–J). The statistical significance of the interaction effect between mouse type (wild‐type vs. cPLA2 KO) and treatment (NF vs. vehicle) on inflammation was determined by two‐way ANOVA (E, F). Quantification of phospho‑p65 was performed as the ratio of phospho‑p65 to total p65, with both signals first normalized to GAPDH (B, E, I). ^∗^ *p* <0.05, ^∗∗^ *p* <0.01, ^∗∗∗^ *p* <0.001, ^∗∗∗∗^ *p* <0.0001, ns, not significant.

To assess whether this anti‑inflammatory effect is dependent on cPLA2, we employed cPLA2 KO mice and cPLA2‑overexpressing HepG2 cells (validation shown in Figure S6A,B). Two‐way ANOVA analysis showed a significant interaction between genotype (WT vs. cPLA2 KO) and treatment (NF vs. vehicle) on inflammation (*p* < 0.05). In WT mice, NF reduced p65 phosphorylation (Figure 6E) and decreased hepatic expression of *Tnf‑α, Il‑1β*, and *Il‑6* (Figure 6F). In HepG2 cells, NF significantly reduced AA release and downstream eicosanoid production (including 12‑HETE and 13‑HODE), as well as PCNA and Ki‑67 expression; these inhibitory effects were attenuated upon cPLA2 overexpression (Figure 6G,H). Furthermore, NF suppressed LPS‑induced p65 phosphorylation (Figure 6I) and downregulated pro‑inflammatory gene expression (*Tnf‑α, Il‑1β, Il‑6*) in control cells, whereas these effects were diminished in cPLA2‑overexpressing cells (Figure 6J). Collectively, these data demonstrate that NF inhibits NF‑κB‑driven inflammation in a cPLA2‑dependent manner.

### 3.7 cPLA2 Activation is Associated With Adverse Clinical Outcome in Human HCC

To assess the clinical relevance of cPLA2 in HCC, we first examined its activation status in human liver tissues. IF staining revealed significantly elevated phosphorylation of cPLA2 in tumor tissues compared with matched adjacent non‐tumor tissues (Figure 7A,B). Consistent with tissue findings, plasma samples from HCC patients exhibited higher cPLA2 enzymatic activity and elevated AA levels compared with healthy controls (Figure 7C). However, we acknowledge that the use of healthy controls for this comparison is a limitation, as cirrhotic or chronic hepatitis samples would provide a more clinically relevant baseline for assessing HCC‑specific alterations. Consistent with Figure S1A, *Pla2g4a* mRNA levels did not differ significantly between normal and HCC patients in the TCGA‑LIHC and GTEx cohort (Figure S7). To further evaluate the relationship between cPLA2 and clinical outcomes, Kaplan‐Meier survival analysis were performed using TCGA‐LIHC data stratified by *PLA2G4A* mRNA expression. Patients with high *PLA2G4A* expression had a significantly shorter disease‑free interval (DFI) than those with low expression. Additionally, a trend toward decreased overall survival (OS) and progression‑free interval (PFI) was observed (Figure 7D–F). Collectively, these findings highlight the clinical relevance of cPLA2 activation in HCC and suggest its potential as a therapeutic target.

**FIGURE 7:**
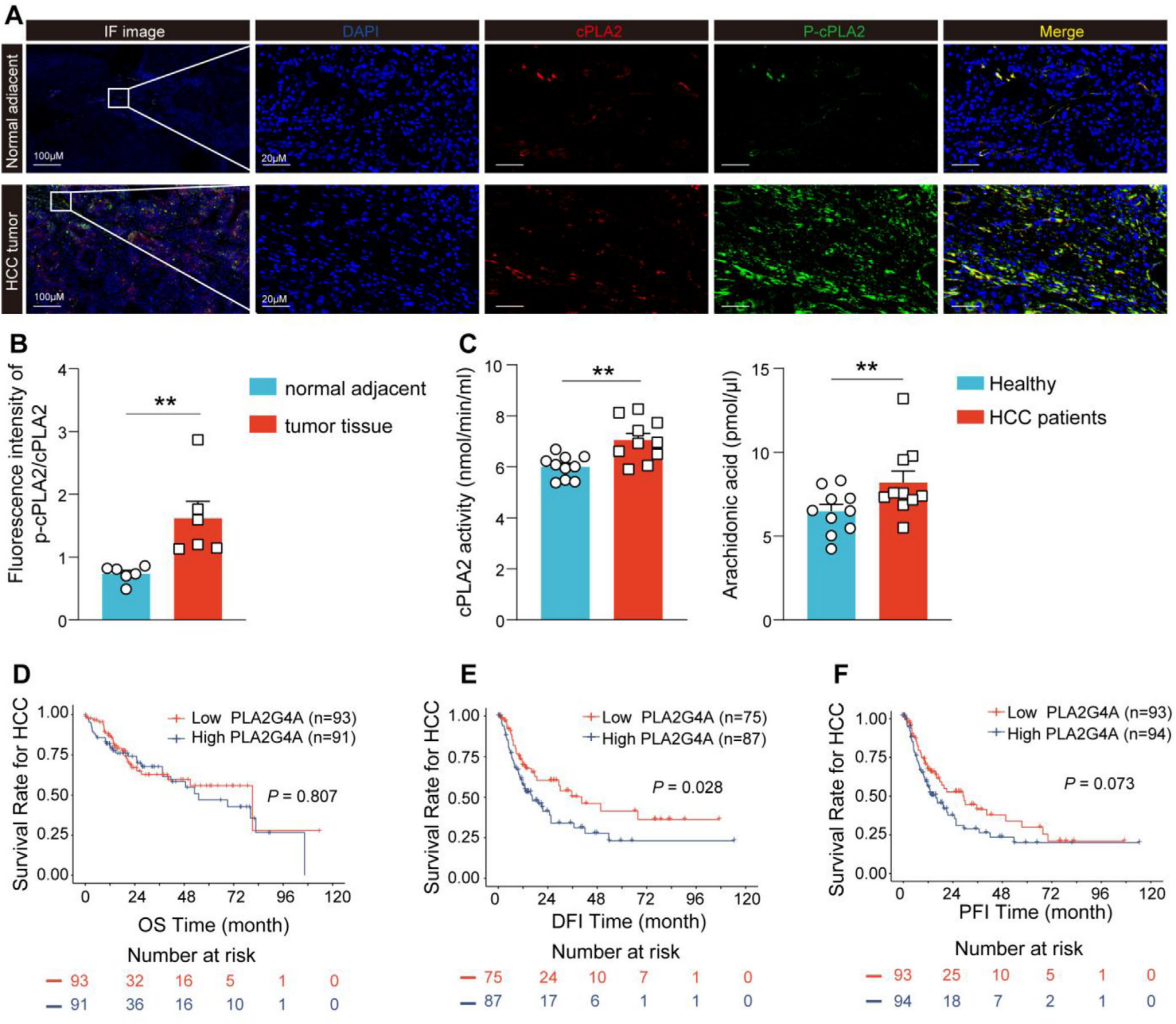
cPLA2 activation is associated with adverse clinical outcomes in human HCC. (A) Representative IF images of phosphorylated cPLA2 in paired tumor (T) and adjacent non‐tumor (NT) liver tissues from patients with HCC. blue, DAPI; red, cPLA2; green, p‐cPLA2. Scale bars, 100/20 µm. (B) Quantified fluorescence intensity of p‐cPLA2/cPLA2 (n = 6). (C) Plasma cPLA2 activity and AA levels in healthy controls and patients with HCC (*n* = 10). (D–F) Kaplan‐Meier survival analyses. For OS (D), DFI (E), and PFI (F) analyses, *PLA2G4A* expression levels were obtained from TCGA‑LIHC tumor samples and normalized as log_2_(TPM + 1). After excluding cases with missing expression values, the OS analysis included 184 liver tumor samples (93 in the low‑expression group and 91 in the high‑expression group); the DFI analysis included 162 liver samples (75 low and 87 high); and the PFI analysis included 187 liver samples (93 low and 94 high). Data are presented as mean ± SEM. Statistical analyses were performed using Mann‐Whitney test (B, C) or log‑rank test (D‐F). D‐F: p values were corrected using the Benjamini‐Hochberg method to control the false discovery rate. ^∗∗^ *p* <0.01.

## 4 Discussion

Chronic liver inflammation is a central driver of hepatocarcinogenesis, yet the upstream molecular mechanisms that sustain inflammatory lipid flux during HCC progression remain incompletely defined. In the present study, we identify enhanced cPLA2 activation‐characterized by increased phosphorylation and membrane recruitment‐as a feature of both murine and human HCC. Genetic ablation of cPLA2 attenuates the inflammatory tumor microenvironment and suppresses hepatocarcinogenesis, establishing cPLA2 as a valuable therapeutic target in HCC. Our data also indicates that pharmacological modulation of cPLA2 activity by NF attenuates inflammatory lipid remodeling and tumor load in vivo. By directly engaging the C2 domain, NF interferes with membrane recruitment of cPLA2 and attenuates inflammatory lipid remodeling, suggesting that membrane recruitment by binding C2 domain of cPLA2 represents a pharmacologically accessible regulatory step in inflammatory HCC (Figure 8).

**FIGURE 8:**
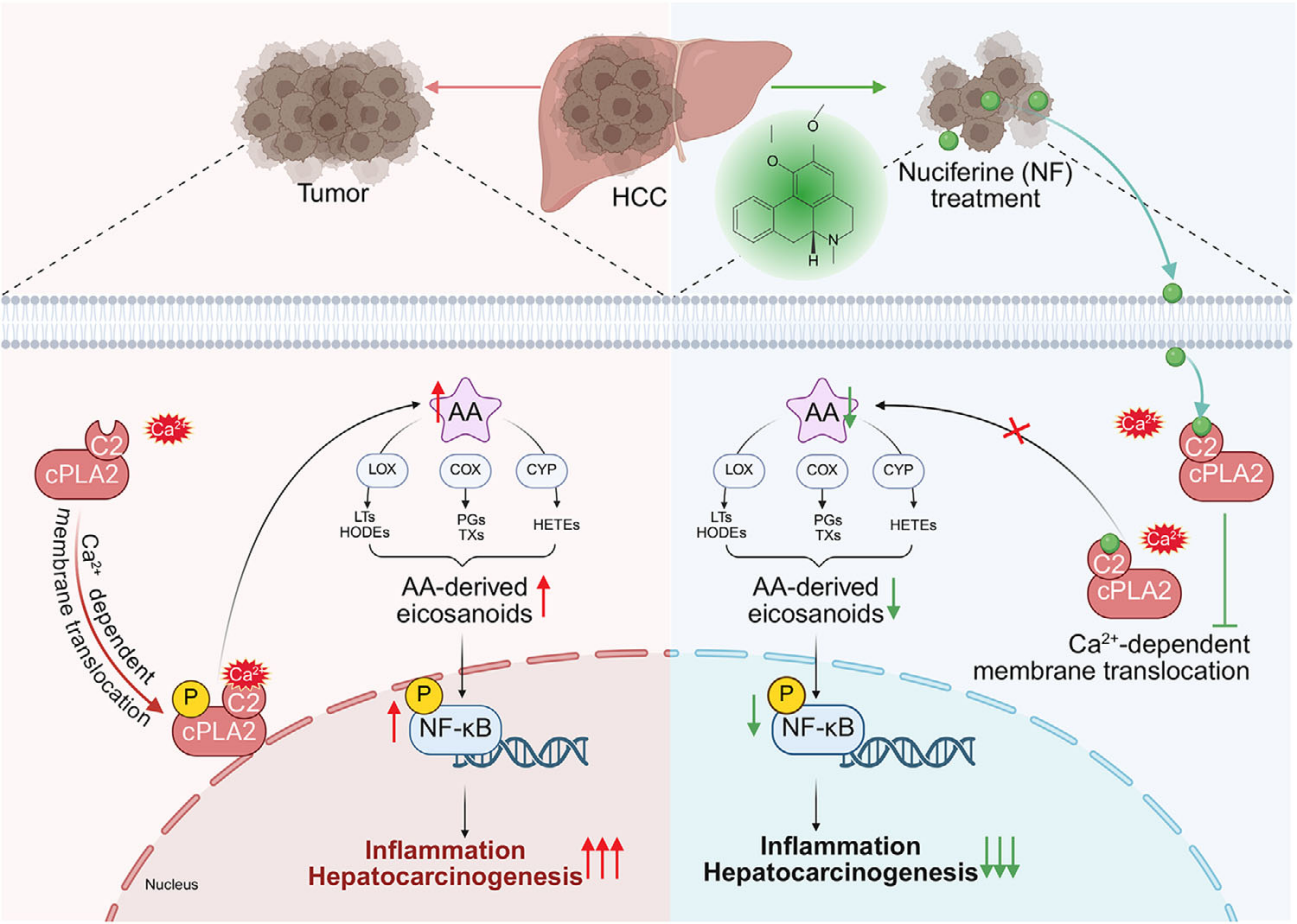
Schematic illustration of the mechanism underlying nuciferine‐mediated inhibition of hepatocellular carcinoma (HCC) progression by targeting cPLA2‐dependent arachidonic acid metabolism. Nuciferine binds to the C2 domain of cPLA2, impairing its Ca^2^^+^‐dependent membrane translocation and enzymatic activation, thereby reducing the release of AA from phospholipids. AA is subsequently metabolized via LOX, COX, and CYP pathways to generate pro‐inflammatory eicosanoids (e.g., LTs, PGs, TXs, and HETEs), which activate NF‐κB signaling and drive inflammatory hepatocarcinogenesis.

Dysregulated AA metabolism has long been implicated in chronic liver injury and tumorigenesis [6, 35]. Elevated levels of AA‐derived eicosanoids promote tumor cell proliferation, angiogenesis, immune modulation, and fibrogenesis, thereby shaping a pro‐tumorigenic microenvironment [36, 37, 38, 39, 40]. Previous therapeutic strategies have largely focused on inhibiting downstream enzymes such as cyclooxygenases or lipoxygenases [41, 42, 43, 44, 45]. However, these approaches may be limited by pathway redundancy and systemic toxicity [46, 47]. Targeting cPLA2 offers an alternative strategy by intervening at the rate‐limiting step of AA liberation [48, 49]. Our results support a model whereby, during DEN/CCl_4_‐induced hepatocarcinogenesis, enhanced cPLA2 activation drives AA‐derived eicosanoid production and fuels a pro‐tumorigenic inflammatory microenvironment. Genetic ablation of *Pla2g4a* markedly attenuated HCC development and suppressed inflammation‐associated pathways in vivo, suggesting that pharmacological modulation of cPLA2 may attenuate inflammatory lipid remodeling more proximally within the signaling cascade.

The C2 domain of cPLA2 mediates Ca^2+^‐dependent docking to perinuclear and endoplasmic reticulum membranes, thereby positioning the enzyme for phospholipid substrate access [13, 50, 51, 52]. While structural studies have established the importance of this domain for enzymatic activation, its relevance as a pharmacological target in cancer has not been fully explored. SPR analysis showed direct binding of NF to the isolated C2 domain with a Kd of 14.62 µM. This binding translated into functional effects in cells, including impaired Ca^2+^‑driven membrane translocation, and was accompanied by conformational changes. These findings support a model in which membrane recruitment represents a druggable regulatory checkpoint in cPLA2 activation. Molecular docking further suggested a potential interaction involving residues near the Ca^2^^+^‑binding loops (e.g., Asn65), although this assignment remains to be experimentally validated by point mutagenesis. Notably, NF binding to the C2 domain also reduced Ser505 phosphorylation (Figure 3B). As molecular docking indicated no direct interaction with the catalytic domain, we interpret this as an indirect effect. NF binding to the C2 domain may induce conformational changes that indirectly affect Ser505 phosphorylation or its functional consequence [53]. Second, NF may primarily impair Ca^2+^‐dependent membrane translocation by targeting the C2 domain, thereby limiting access of cPLA2 to membrane phospholipid substrates [17, 54, 55, 56]. Consistent with this, published pharmacokinetic data indicate that after a single oral dose of 15 mg/kg NF in mice, hepatic homogenate concentrations reach approximately 68–81 µM at 0.5 h post‑administration, a value that is about 4.6‑ to 5.6‑fold above the SPR‑derived Kd of 14.62 µM [57]. Although this estimate derives from a slightly higher dose than the 10 mg/kg used in our study and may not reflect the free intracellular concentration, it provides an order‑of‑magnitude basis for considering target engagement in vivo.

To further clarify the functional relevance of cPLA2 in vivo, we examined the therapeutic response to NF in cPLA2 KO mouse models. Genetic ablation of cPLA2 significantly suppressed HCC development in mice, and the antitumor effect of NF observed in wild‐type mice was largely abrogated in the absence of cPLA2 (Figure 5B). These findings are consistent with a cPLA2‑dependent therapeutic response to NF. However, a floor effect cannot be excluded, as the tumor burden in knockout mice may already be too low for any additional NF‑attributable reduction to be statistically detectable. Additionally, because cPLA2 is abundantly expressed not only in hepatocytes but also in macrophages and neutrophils [58], the global KO model cannot distinguish hepatocyte‐intrinsic effects from those mediated by immune cells. A hepatocyte‑specific cPLA2 KO model will therefore be required to define the cell‐type‐specific mechanism underlying NF activity. cPLA2 is required for the full therapeutic effect of NF, although the possible involvement of other molecular targets cannot be formally excluded. Our data support a model in which cPLA2‐dependent lipid signaling constitutes a modulatory axis that can be pharmacologically engaged to restrain tumor load.

Downstream of AA release, we observed attenuation of NF‐κB signaling following NF treatment, consistent with reduced expression of pro‐inflammatory cytokines. Given the established role of NF‐κB in chronic liver injury and tumor progression, suppression of this pathway likely contributes to the antitumor phenotype observed in our models [59, 60, 61]. Importantly, cPLA2 overexpression markedly attenuated the inhibitory effects of NF on AA release and lipid mediator production, indicating that NF suppresses AA metabolism and related inflammatory lipid signaling in a cPLA2‑dependent manner. Notably, the inhibitory effect of NF on NF‐κB activation was diminished in cPLA2‐deficient settings as well as in cPLA2‐overexpressing cells, further supporting that modulation of inflammatory transcriptional programs occurs, at least in part, through cPLA2‐dependent lipid signaling [62].

Several limitations of the present study should be acknowledged. The clinical analysis of cPLA2 activation was based on a relatively limited number of tissue specimens, primarily owing to the availability of clinical materials and the constraints of the sample collection schedule. Larger and independent clinical cohorts will therefore be required to confirm the association between cPLA2 activation and HCC progression. Future studies incorporating larger clinical cohorts, relevant translational models, and, when available, clinical intervention samples will be necessary to further establish the therapeutic relevance of targeting cPLA2 with NF in human HCC.

In summary, our study demonstrates that cPLA2 activation is enhanced during hepatocarcinogenesis and can be pharmacologically modulated through interference with its C2 domain‐mediated membrane recruitment. NF directly engages the C2 domain of cPLA2 and interferes with Ca^2+^‐dependent membrane recruitment, revealing membrane translocation as a pharmacologically accessible regulatory checkpoint in cPLA2 activation. The attenuation of hepatocarcinogenesis in *Pla2g4a* KO mice, together with the loss of NF efficacy in the absence of cPLA2, further supports cPLA2 as a principal mediator of the antitumor activity of NF. Consistent with these experimental findings, enhanced cPLA2 activation and dysregulated AA metabolism in human HCC underscore the potential clinical relevance of this inflammatory lipid axis. Collectively, our findings establish cPLA2 as a therapeutically actionable regulator of AA‐dependent inflammatory flux and suggest that targeting upstream AA liberation may provide a complementary strategy for restraining inflammation‐driven HCC.

## Author Contributions

J. S., J. D. and H. Y. designed the experiments. J. S., Y. L. and X. F. performed the experiments. J. S., X. W., S. L. and W. W. analyzed and interpreted the data. X.W., T. Z., J. L., D. P. and H. Y. provided technical support. J. L. provided human samples. J. S., J. D. and H. Y. wrote the manuscript. X. L., and H. Y. supervised the study. All authors critically read and commented on the final manuscript.

## Funding

This work was financially supported by National Natural Science Foundation of China (32422069) and the 2115 Talent Development Program of China Agricultural University.

## Conflicts of Interest

The authors declare no conflicts of interest.

## Supporting information




**Supporting File 1**: advs78212‐sup‐0001‐SuppMat.doc.

**Supporting File 2**: advs78212‐sup‐0002‐SuppMat.csv.

## Data Availability

The data that support the findings of this study are available from the corresponding author upon reasonable request.
