# Supplementary material for: Nuciferine Suppresses Inflammatory Hepatocarcinogenesis by Targeting cPLA2‐Dependent Arachidonic Acid Metabolism
Source: Adv Sci (Weinh). 2026 Oct 8:e78212. Online ahead of print. doi: 10.1002/advs.78212 (PMC13650035; doi:10.1002/advs.78212)
Supplement: Supplementary file 1 [file ADVS-9999-e78212-s002.doc]

**Nuciferine suppresses inflammatory hepatocarcinogenesis by targeting cPLA2-dependent arachidonic acid metabolism**

Jing Sun^a^, Xinyan Wu^a^, Shuwei Luo^a^, Yilang Liu^a^, Jie Liu^b^, Jun Lu^c^, Weimin Wang^a^, Xiao Feng^a^, Tuo Zhang^a^, Dipak Panigrahy^d^, Jianjun Deng^e^, Xiaojun Liao^a,*^, Haixia Yang^a,*^

^a^ College of Food Science and Nutritional Engineering, China Agricultural University, Beijing 100083, China

^b^ Department of General Surgery, Dazhou Central Hospital, Dazhou 635000, China

^c^ Department of General Surgery, Peking University Third Hospital, Beijing 100191, China

^d^ Center for Vascular Biology Research, Beth Israel Deaconess Medical Center, Harvard Medical School, Boston MA 02215, U.S.A

^e^ State Key Laboratory of Vegetable Biobreeding, Institute of Vegetables and Flowers, Chinese Academy of Agricultural Sciences, Beijing 100081, China

*Corresponding author: Haixia Yang (hyang@cau.edu.cn), Xiaojun Liao (liaoxjun@cau.edu.cn)

**Table of contents**

**Supplementary Materials and Methods**	2

**Supplementary Figures**	11

**Supplementary Tables**	16

**Supplementary References**	22

### **Supplementary Materials and Methods**

**Ethics statement**

All animal experiments were conducted in accordance with institutional guidelines and were approved by the Institutional Animal Care and Use Committees of China Agricultural University (approval no. AW12902202-4-1). All procedures involving human samples were approved by the Medical Ethics Committee of Dazhou Central Hospital (approval no.  2025186) and complied with relevant ethical guidelines and regulations. Written informed consent was obtained from all participants prior to sample collection.

**Human samples**

Paired formalin-fixed paraffin-embedded (FFPE) sections of hepatocellular carcinoma (HCC) and adjacent non-tumor liver tissues were analyzed by immunofluorescence staining. Blood samples were obtained from a cohort of patients diagnosed with HCC or liver cirrhosis who had no prior history of other malignancies. Samples from healthy volunteers, confirmed to be free of any medical abnormalities through routine examination, served as controls. Clinicopathological details are provided in Table S1. All blood samples were immediately frozen in liquid nitrogen upon collection and stored at -80°C until further analysis.

To perform Kaplan-Meier analysis based on TCGA-LIHC datasets, we used web tools of Sparkle (<https://grswsci.top/>). *PLA2G4A* expression data and corresponding clinical follow‑up information for LIHC tumor samples were retrieved from the TCGA database, with only tumor tissues included and cases with missing expression values excluded. For all three survival endpoints (OS/DFI/PFI), samples were stratified by *PLA2G4A* expression quartiles determined in the full tumor cohort prior to endpoint‑specific filtering (R quantile function, default type‑7 algorithm), generating four groups: Q1 (>75%, high expression), Q2 (50-75%), Q3 (25-50%), and Q4 (≤25%, low expression). For each endpoint, cases with missing or non‑positive survival time (≤ 0 years) or missing event status were excluded. To more intuitively illustrate the association between *PLA2G4A* expression and patient prognosis, only the extreme quartiles (Q1 and Q4) are displayed for comparison. Survival differences between groups were assessed using the log‑rank test, with *P* values adjusted by the Benjamini-Hochberg method for multiple comparisons. Endpoint-specific optimal cutoffs for *PLA2G4A* expression were determined using the maximal standardized log‑rank statistic implemented in the surv_cutpoint function of the survminer R package, with a minimum group proportion of 20%.

**Animals and housing**

Male C57BL/6 mice (3 weeks old) were obtained from SPF Biotechnology Co. Ltd., and cPLA2 heterozygous mice (cPLA2^+/−^) (6 weeks old) were obtained from GemPharmatech (Nanjing, China). Mice were housed under specific pathogen-free (SPF) conditions with a 12 h light/12 h dark cycle at 18-22 °C and 50-65% humidity, with ad libitum access to food and water. Mice were randomly assigned to experimental groups. All animal experiments were approved by the Institutional Animal Care and Use Committee (IACUC) and performed in accordance with the principles of the 3Rs (Replacement, Reduction, and Refinement), with the aim of ensuring sufficient statistical power to detect biologically relevant effects while minimizing animal use.

**Effects of NF on DEN/CCl_4_-induced HCC mice**

A DEN/CCl_4_-induced HCC model was established in male C57BL/6 mice using a modified version of a previously described protocol.^[1]^ At 3 weeks of age, mice received a single intraperitoneal (i.p.) injection of DEN (25 mg/kg body weight; Sigma-Aldrich, St. Louis, MO). From 9 to 27 weeks of age, mice were administered i.p. injections of CCl_4_ (0.5 mL/kg body weight, diluted in olive oil; Macklin, C805325, Shanghai, China) twice weekly. Concurrently, from 7 to 27 weeks of age, mice were treated daily by oral gavage with either NF (10 mg/kg body weight; Biopurify, BP1014, Chengdu, China) dissolved in 0.5% carboxymethyl cellulose sodium (CMC-Na) or vehicle (0.5% CMC-Na) alone. Control mice received equivalent volumes of PBS or olive oil at the corresponding time points. All tumor burdens remained within ethical limits (<10% body weight, diameter <20 mm). At the experimental endpoint, mice were sacrificed. Blood and liver tissues were collected for subsequent analyses.

**Effects of NF on DEN/CCl_4_-induced hepatocarcinogenesis in cPLA2 KO mice**

To investigate the role of cPLA2 in NF-mediated hepatoprotection, homozygous cPLA2 KO (cPLA2^⁻/⁻^) and wild-type (cPLA2^⁺/⁺^) male littermates generated from heterozygous crossings were subjected to the DEN/CCl_4_-induced HCC model as described above. Throughout the experiment, mice received either NF or vehicle following the same dosing regimen.

**AST and ALT measurements**

Plasma was isolated by centrifugation of EDTA-anticoagulated blood at 3,000 × g for 10 min at 4 °C. Plasma ALT and AST levels were measured using commercial assay kits (Nanjing Jiancheng, C009-2-1 and C010-2-1, China) according to the manufacturer’s instructions.

**Pro-inflammatory cytokines measurements**

Pro-inflammatory cytokines (IL‑1β, IL‑6, and TNF‑α) were quantified using ELISA kits (Fankew, F30626‑A, F30717‑B, F2132‑A) according to the manufacturers’ instructions.

**Histology analysis**

Liver tissues were fixed in 4% paraformaldehyde (Wuhan servicebio technology, CR2109113) for 24 h, paraffin-embedded, sectioned (5 μm) and stained with hematoxylin and eosin (H&E) (Servicebio, G1004) or Sirius Red (Servicebio, GP2089). Histological damage was scored based on tissue structure and fibrosis under an optical microscope (NIKON ECLIPSE E100, Nikon Corporation).

**Immunohistochemistry**

After antigen retrieval, liver tissues were blocked with 5% bovine serum albumin for 30 min and incubated overnight at 4 °C with primary antibodies against Ki67 (1:800, Servicebio, GB121141) or proliferating cell nuclear antigen (PCNA, 1:1000, Servicebio, GB12010). Sections were then incubated with a biotinylated secondary antibody for 50 min at room temperature, developed with DAB (Vector Laboratories), and counterstained with hematoxylin. Images were acquired using an optical microscope (Nikon ECLIPSE E100, Nikon Corporation).

**Immunofluorescence**

Paraffin-embedded liver tissues from human or mouse specimens were dewaxed, rehydrated, and subjected to antigen retrieval. Sections were incubated overnight at 4 °C with primary antibodies against cPLA2 (1:1000, Abmart, PA3288) or phosphorylated cPLA2 (1:1000, Abmart, PA3287). After washing, sections were incubated with HRP-conjugated goat anti-rabbit secondary antibodies (Servicebio), followed by counterstaining with DAPI (0.5 µg/mL). Stained sections were imaged using an Imager A2 Axio upright microscope (Zeiss).

**RNA extraction and quantitative Real-time PCR (RT-qPCR) analysis**

Total RNA was isolated from liver tissues or cultured cells using TRIzol reagent (Vazyme, R401-01). RNA purity and concentration were determined spectrophotometrically (NanoDrop, Thermo Scientific). Reverse transcription was performed with 1 μg RNA using the HiScript Reverse Transcriptase kit (Vazyme, R433-01). Quantitative PCR was carried out on a Real-Time PCR system (Thermo Fisher Scientific) with SYBR qPCR Master Mix (Vazyme, Q412-02). Primers used for RT-qPCR in this study were listed in the Table S2. 2^-ΔΔCt^ method was used to calculate the fold change in gene expression.

**Targeted LC-MS/MS lipidomic analyses**

Approximately 200 mg of liver tissue was homogenized in an extraction solution containing 0.1 % butylhydroxytoluene, tert‑butylhydroxyanisole, formic acid, and mixed internal standards. After vortexing, samples were shaken at 1500 rpm for 12 h at 4 °C and centrifuged at 12000× g for 10 min (4 °C). The supernatant was subjected to solid‑phase extraction (Oasis Prime HLB column, 30 mg, Waters) to enrich AA and eicosanoids. Extracts were analyzed using an Exion AD30‑UPLC‑Sciex 650 Plus Qtrap system equipped with a Phenomenex Kinetex‑C18 column (100 × 2.1 mm, 1.7 µm). Mobile phases consisted of (A) water: acetonitrile:formic acid (63:37:0.02, v/v/v) and (B) acetonitrile: isopropanol (1:1, v/v). Eicosanoids were identified by comparison with reference standards and quantified using calibration curves. A total of 43 eicosanoids were detected in liver samples (raw LC‑MS/MS data are provided in Table S3). The “NF‑reversed HCC eicosanoids” was defined as eicosanoids that were significantly upregulated in Model vs. Ctrl (adjusted *P* < 0.05, fold change > 1.5) and downregulated in NF vs. Model (adjusted *P* < 0.05, fold change > 1.5). AA levels in human blood samples were profiled using the same targeted lipidomics approach. Among the metabolites quantified, data for AA are presented in this study.

**RNA sequencing analysis**

Total RNA was extracted from liver tissues for RNA sequencing. cDNA libraries were prepared and sequenced by Majorbio Biotech (Shanghai, China). Transcript abundance was quantified as transcripts per million (TPM) using the Ballgown R package. Differential expression analysis across the Ctrl, Model, and NF group was performed with DESeq2 (v1.30.0) in R (v4.0.3), and ‘NF‑reversed HCC geneset’ are defined as significantly upregulated in Model vs. Ctrl (adjusted *P* < 0.05, fold change > 1.5) and downregulated in NF vs. Model (adjusted *P* < 0.05, fold change > 1.5). Gene Set Enrichment Analysis (GSEA) was conducted using GSEAPreranked tool (v4.1.0), in which genes were preranked by *P* value and fold change. Gene Ontology (GO) enrichment analysis was carried out with clusterProfiler, applying a significance threshold of *P* < 0.05.

**cPLA2 activity measurements**

cPLA2 activity was measured in mouse plasma, liver homogenates, and human blood samples using a commercial ELISA kit (Cayman, 765021). Liver tissues were homogenized in PBS (40 mg/200 μL), followed by centrifugation at 10,000 × g for 15 min at 4 °C. The resulting supernatant and plasma samples were processed according to the manufacturer’s protocol.

**Western blot analysis**

Total protein was extracted with RIPA lysis buffer (solarbio, R0020) with protease/phosphatase inhibitor cocktail (Beyotime Biotechnology, P1045). Protein concentrations were quantified using a BCA protein kit (solarbio, PC0020) on supernatants of centrifuged samples. The protein samples were denatured at 98°C for 5 min before SDS-PAGE and were electroblotted into PVDF membrane (millipore) electrophoretically using a semi-dry transfer system (Bio-Rad, Trans-Blot^®^ Turbo^™^). The membrane was blocked using 5% (w/v) non-fat milk in TBST for 1h at room temperature, followed by incubating with primary antibody at 4°C overnight and further incubated with secondary antibody at room temperature for 1 h. The immunogenic bands were developed by chemiluminescent (ECL) substrate (Bioprimacy) and visualized by the Tanon 4600 system. The following primary and secondary antibodies were used: GAPDH (Beyotime Biotechnology, AF1186, 1:3000), anti-cPLA2 (Abmart, PA3288S, 1:500), anti-p-cPLA2 (Ser505) (Abmart, PA3287, 1:500), anti-NF-κB p65 (Cell signaling Technology, 8242, 1:1000), anti-p-NF-κB p65 (Cell signaling Technology, 3033, 1:1000), anti-Lamin B1 (Abmart, P60054F, 1:1000), Goat anti-rabbit-IgG(H+L) (Beyotime Biotechnology, 1:3000). Quantification was performed with biological replicates (n ≥ 3 independent experiments).

**Native-PAGE analysis**

The target protein was mixed thoroughly with Native loading buffer (10 mM Tris-HCl, 1 mM EDTA, and 0.002% bromophenol blue) without heating. Electrophoresis was performed on a 4-20% polyacrylamide gradient gel at a constant voltage of 180 V for 70 min, with the entire process conducted at 4°C under cold-cycling conditions.

**Cell culture experiments and treatments**

RAW 264.7 cells (CRL-2278) and HepG2 cells (HB-8065) were obtained from the American Type Culture Collection (ATCC, Manassas, VA, USA). Both cell lines were cultured in Dulbecco’s modified Eagle’s medium (DMEM; Gibco, GNM-12850). All culture media were supplemented with 10% fetal bovine serum (Pricella, 164220) and 1% penicillin–streptomycin (Gibco, sNA-001). Cells were maintained at 37 °C in a humidified incubator with 5% CO_2_. Cells were passaged using 0.05 % trypsin (Gibco, 25300054).

**Drug affinity responsive target stability (DARTS) measurements**

The DARTS method has been described previously and had some modifications.^[2]^ Briefly, RAW264.7 cells were lysed using M-PER™ Mammalian Protein Extraction Reagent (Thermofisher, 78501), followed by centrifugation at 12,000×g for 10 min at 4°C. The resulting cell lysates were incubated with varying concentrations of NF (10, 30, 90 µM) or equivalent volumes of dimethyl sulfoxide (DMSO) for 2 h at 37°C with shaking at 100 rpm. Subsequently, samples were digested with pronase (1:600 (w/w), 200 µg/mL) for 15 min at 37°C. The digestion was halted by boiling the samples in protein loading buffer for subsequent western blot analysis.

**Cellular thermal shift assay (CETSA) measurements**

The CETSA was performed according to established protocols and had some modifications.^[2]^ Briefly, RAW264.7 and HepG2 cells were treated with NF (30 µM) or equivalent DMSO for 1 h at 37°C and subsequently washed three times with PBS, separately. The cells were then suspended in PBS and divided equally into multiple sterile Eppendorf tubes, followed by heating for 3 min at various temperatures (40, 43, 46, 49, 52, 55, 58, 61, 64 °C). After heating, three snap freeze-thaw cycles were applied to lyse the cells, and the samples were centrifuged at 12,000× g for 15 min at 4°C. The supernatants were boiled and collected for western blot analysis.

**Ca^2+^-driven cPLA2 membrane translocation measurements**

RAW 264.7 and HepG2 cells grown in 6‑well plates were treated with NF (30 μM) or vehicle for 12 h, then stimulated with 1 μM Ca^2+^ (Beyotime Biotechnology, S1672) for 5 min. Cells were lysed using a Nuclear and Cytoplasmic Protein Extraction Kit (Beyotime Biotechnology, P0027). Cytosolic and nuclear fractions were collected to evaluate cPLA2 concentration.

**Plasmids extraction and cell transfection**

HepG2 cells were transfected with a cPLA2 expression plasmid (MiaoLing Biotechnology, P38520) or the corresponding empty vector using Lipo8000™ Transfection Reagent (Beyotime Biotechnology, C0533), according to the manufacturer’s instructions. At 24 h post-transfection, cells were treated with NF (30 μM) or vehicle for the indicated durations. Cells were harvested for RNA and protein extraction to evaluate cPLA2 expression and downstream signaling components. Besides, concentration of AA and eicosanoids, including 12-hydroxyeicosatetraenoic acid (12-HETE) and 13-hydroxyoctadecadienoic acid (13-HODE) were measured using commercial assay kits (Shanghai mlbio, China) according to the manufacturer’s instructions.

**cPLA2 expression and purification**

The plasmid pT7-6×His-*PLA2G4A* (MiaoLing Biotechnology, P38520) encoding human cPLA2 was transformed into *E. coli* Rosetta (DE3) cells (Weidi Biotechnology, EC1010). Protein expression was induced with 0.2 mM IPTG when the culture reached an OD_600_ of 0.6-0.8, followed by incubation at 20 °C for 12-16 h. cPLA2 was expressed predominantly in inclusion bodies, which were denatured in 8 M urea buffer (20 mM Tris‑HCl, 200 mM NaCl, 1 mM EDTA, 5 mM Tris(2-carboxyethyl)phosphine (TCEP), pH 8.0). Refolding was performed by stepwise dialysis against decreasing urea concentrations (6, 4, 2, 1, 0.5 M) over 24 h. The refolded protein was purified using Ni‑NTA agarose (Invitrogen, R90101) and concentrated to 1 mg/mL in 20 mM Tris‑HCl (pH 8.0) containing 200 mM NaCl using a 10 kDa centrifugal filter (Millipore).

**cPLA2 C2 domain expression and purification**

Expression and purification of the cPLA2 C2 domain protein were performed as described.^[3]^ The C2 domain (residues 17-140) of human cPLA2 was expressed as an N‑terminal 6×His‑SUMO fusion protein from a pET‑SUMO vector in *E. coli* Rosetta (DE3) cells. Expression was induced with 0.1 mM IPTG at an OD_600_ of 0.6-0.8, followed by growth for 16 h at 20 °C. Cells were harvested, resuspended, and lysed by sonication. After centrifugation, the soluble fraction was loaded onto Ni‑NTA agarose (Invitrogen, R90101). The bound fusion protein was eluted, concentrated, and treated with Ulp1 protease (Beyotime Biotechnology, P2312S) to remove the His‑SUMO tag. The cleaved product was reapplied to Ni‑NTA resin to remove the protease and uncleaved fusion protein. Final purification was performed using an ÄKTA Prime system equipped with a HiTrap Q HP anion‑exchange column (1 mL) in 20 mM Tris‑HCl (pH 8.0) containing 2.5 mM CaCl_2_.

**Surface plasmon resonance (SPR) measurement**

Binding affinity between NF and the recombinant human C2 domain of cPLA2 was measured by SPR on a Biacore T200 instrument (Cytiva) using CM5 sensor chips. The C2 domain (0.6 mg/mL in PBS) was immobilized on the chip via amine coupling, yielding a density of ~3700 RU. Serial dilutions of NF (0-160 μM, containing 3 % DMSO) were injected over the chip surface. Binding kinetics were analyzed with Biacore T200 Evaluation Software (v3.0, Cytiva), and dissociation constants (K_D_) were derived. Sensorgrams were plotted using GraphPad Prism 9.0.

**Fluorescence titration**

Fluorescence measurements were carried out on a Cary Eclipse spectrophotometer (Varian).^[4]^ Full‑length cPLA2 or its isolated C2 domain (1.0 μM in 10 mM Tris‑EDTA, pH 8.0) was titrated with successive 1 μL aliquots of NF stock solution (1.0 mM in DMSO). Emission spectra (290-500 nm) were recorded after excitation at 280 nm, with slit widths set to 10 nm (excitation) and 5 nm (emission). To monitor ligand‑induced conformational changes, synchronous fluorescence spectra were acquired at wavelength intervals (Δλ) of 15 nm (tyrosine, Tyr) and 60 nm (tryptophan, Trp) for both protein constructs.

The binding constant K and the binding number n was calculated according to the equation (1):

I_0_: the fluorescence intensity of cPLA2, I_∞_: the fluorescence intensity at cPLA2 binding site saturation, n: binding number, [P]_0_: concentration of cPLA2, [NF]_0_: concentration of NF, K: binding constant.

The quenching constant K_q_ can be calculated from the slope of the linear Stern-Volmer plot:

F_0_: fluorescence intensity of cPLA2, F: fluorescence intensity of cPLA2 with NF, K_q_: Quenching Constant of cPLA2, τ_0_: the average fluorescence lifetime of proteins without quenchers; for tryptophan residues in proteins, it is approximately 10^-9^ s, [Q]: the concentrations of NF, K_sv_: Dynamic Quenching Constant.

**Circular Dichroism (CD) spectra measurement**

CD spectra were acquired using a Chirascan spectrometer (Applied Photophysics, UK). Measurements were performed on full-length cPLA2 and its C2 domain at concentrations of 1.0 μM and 20 μM, respectively, in 25 mM phosphate buffer (pH 8.0). A stock solution of NF was prepared in acetonitrile. Spectra represent the average of three consecutive scans recorded from 190 to 260 nm at 100 nm/min, with a sensitivity of 1 mdeg, a bandwidth of 1.0 nm, a resolution of 0.5 nm, and an integration time of 2 s. Buffer baselines were subtracted, and raw ellipticity (mdeg) was converted to mean residue ellipticity (θ, deg cm^2^ dmol^-1^). Secondary structure content was quantified using the CDNN software.

**Competitive Ca^2+^-binding assay**

The purified C2 domain (20 µM) was placed in pre‑equilibrated dialysis tubing and dialyzed against 2 mM HEPES buffer (pH 8.0) containing 5 mM CaCl_2_. Dialysis was performed with continuous stirring at 4°C for 1 h, with the dialysis buffer replaced three times to ensure sufficient equilibration of the C2 domain with Ca^2+^. To remove unbound Ca^2+^, the dialysis tubing was then transferred to Ca^2+^‑free HEPES buffer and subjected to three additional rounds of dialysis with buffer changes. The Ca^2+^‑bound C2 domain was recovered and divided into two equal aliquots. One aliquot was incubated with NF at a final concentration of 100 µM, and the other received an equal volume of solvent control. Both samples were incubated at 30 °C for 30 min. Following incubation, the samples were subjected to ultrafiltration, and the filtrates were collected for Ca^2+^ content analysis using inductively coupled plasma mass spectrometry (ICP‑MS; iCAP 7400, Thermo Fisher Scientific, Waltham, MA, USA). All experiments were performed with biological replicates (n = 3 independent experiments).

**Statistical analysis**

Data were analyzed using GraphPad Prism 9.0 and are presented as mean ± SEM unless otherwise stated. All experiments were performed with biological replicates (n ≥ 3 independent experiments). Statistical comparison of two groups was performed using either Student’s t-test or Mann-Whitney test, and comparison of three groups was analyzed by one-way ANOVA. In the cPLA2 KO mouse experiments (Fig. 5 and 6), two-way ANOVA was applied to assess the effects of NF treatment. Survival data from TCGA‑LIHC were evaluated with the log‑rank test. A *P* value < 0.05 was considered statistically significant. Further details are provided in the figure legends.

**Supplementary Figures**

**Figure S1. Biochemical analysis of the interaction between NF and cPLA2.** (A) Hepatic *Pla2g4a* (cPLA2) mRNA levels determined by RNA-Seq and RT-qPCR (n=6 mice/group); (B) CETSA assessing the thermal stabilization of cPLA2 by NF in HepG2 cell lysates (40-64 °C). GAPDH was used as a loading control. Data are presented as mean ± SEM and representative of 3 independent biological replicates. Statistical analyses were performed by one-way ANOVA. ∗*P* <0.05, ns, not significant.

**Figure S2. cPLA2 identification and spectral analysis of the interaction between NF and cPLA2.** (A) SDS-PAGE image of purified cPLA2 protein. (B) AKTA purification chromatogram of recombinant cPLA2 protein, showing two elution peaks (peak 1 and 2). The corresponding fractions were analyzed by native PAGE and western blotting to identify the protein species. (C) cPLA2 activity of the two eluted fractions (peak 1 and peak 2). (D) Fluorescence spectra of purified cPLA2 in the presence of solvent (DMSO). (E) Emission fluorescence spectrum of cPLA2 and ultraviolet absorption spectrum of NF showing spectral overlap consistent with possible FRET. (F) Synchronous fluorescence spectroscopy (△λ= 60 nm) monitoring tryptophan (Trp) residues of cPLA2 in the presence of increasing concentrations of NF or solvent (DMSO) (G) Synchronous fluorescence spectroscopy (△λ= 15 nm) monitoring tyrosine (Tyr) residues of cPLA2 in the presence of increasing concentrations of NF or solvent (DMSO). (H) CD spectra of cPLA2 in the presence of solvent (acetonitrile). Data are presented as mean ± SEM. Statistical analyses were performed using one-way ANOVA.

**Figure S3. Purification of the C2 domain of cPLA2 and analysis of its interaction with NF.** (A) SDS-PAGE image of the purified cPLA2 C2 domain (17-140 AA). Sample 1: C2 domain containing an N-terminal 6xHis-SUMO tag; Sample 2: C2 domain after removal of the N-terminal 6xHis-SUMO tag. (B) AKTA size-exclusion chromatography profile and SDS-PAGE analysis of the purified C2 domain (14 kDa). (C) Western blotting analysis of the cPLA2 C2 domain. (D) Fluorescence spectra of purified C2 domain in the presence of solvent (DMSO). (E) Emission fluorescence spectrum of C2 domain and ultraviolet absorption spectrum of NF showing spectral overlap consistent with possible FRET. (F) Synchronous fluorescence spectroscopy (left: △λ= 15 nm; right: △λ= 60 nm) of C2 domain in the presence of solvent (DMSO). (G) Subcellular localization of cPLA2 in HepG2 cells following Ca^2+^ stimulation with or without NF treatment (n=3 independent biological replicates). Cytosolic and nuclear fractions were analyzed by western blotting. GAPDH and Lamin B1 served as cytosolic and nuclear markers, respectively.

**Figure S4. Generation of global cPLA2 KO mice.** (A). Schematic illustration of the breeding strategy used to obtain homozygous wild-type (cPLA2^+/+)^ and knockout (cPLA2^−/−^) mice by crossing heterozygous (cPLA2^+/−^) mice. (B) Genotyping results showing the 298-bp band for wild-type mice, the 243-bp band for knockout mice, and both bands for heterozygous mice. (C) Western blot analysis of liver lysates from WT and cPLA2 KO mice confirming loss of cPLA2 expression. Data are presented as mean ± SEM and representative of 3 independent biological replicates. *P* value was calculated using Student’s t‑test.

**Figure S5.** GSEA analysis between Ctrl, Model and NF group. (A) GSEA showing upregulation of TNF-α/NF-κB signaling in Model group (n=6 mice/group). (B) GSEA showing downregulation of TNF-α/NF-κB signaling in NF group (n=6 mice/group).

**Figure S6. Validation of cPLA2 overexpression in HepG2 cells.** (A) mRNA expression of *PLA2G4A* in HepG2 cells. (B) Western blot analysis of cPLA2 in HepG2 cells transfected with the corresponding empty vector (NC) or a cPLA2 expression plasmid (OE). Data are presented as mean ± SEM and representative of 3 independent biological replicates. *P* values were calculated using Student’s t‑test.

**Figure S7.** Expression level of *PLA2G4A* in normal and HCC samples based on TCGA-LIHC and GTEx database. All expression data were extracted and processed using the GEPIA2 platform (http://gepia2.cancer-pku.cn/#index), and the values are presented as log2(TPM + 1) normalized expression.

### **Supplementary Tables**

Table S1. Clinicopathological characteristics of HCC patients.

| Sample | Age | Sex | Etiology | Stage | Treatment status |
| --- | --- | --- | --- | --- | --- |
| HCC | 74 | male | - | T1N0M0 | interventional therapy combined with chemotherapy |
|  | 54 | male | - | T4N0M0 | surgery |
|  | 60 | female | - | T3N0M0 | symptomatic therapy |
|  | 74 | female | - | T3N1M0 | symptomatic therapy |
|  | 55 | male | - | T2N1M1 | transcatheter embolization and hepatic arterial infusion chemotherapy |
|  | 70 | female | hepatitis B | T2N1M1 | symptomatic therapy |
|  | - | male | HCC; chronic hepatitis B; hypertension | - | - |
|  | - | male | HCC (postoperative, with multiple metastases) | - | - |
|  | - | male | space‑occupying lesion of the liver | - | - |
|  | - | male | HCC | - | - |
|  | - | male | Hepatic encephalopathy; HCC | CNLC IIIb | - |
|  | - | male | HCC with multiple metastases | - | - |
|  | - | female | HCC | CNLC IIIa | - |
|  | - | female | - | - | surgery |
|  | - | male | decompensated liver cirrhosis | - | surgery |
|  | - | male | chronic hepatitis B | - | - |
| Healthy | - | male | - | - | normal physical examination |
|  | - | male | - | - | normal physical examination |
|  | - | male | - | - | normal physical examination |
|  | - | male | - | - | normal physical examination |
|  | - | male | - | - | normal physical examination |
|  | - | male | - | - | normal physical examination |
|  | - | male | - | - | normal physical examination |
|  | - | female | - | - | normal physical examination |
|  | - | female | - | - | normal physical examination |
|  | - | female | - | - | normal physical examination |

TNM, international cancer staging system (Tumor-Node-Metastasis); CNLC, China liver cancer staging system for HCC.

Table S2. Primer sequences used for RT-qPCR.

| **Species** | **Primer** | | **Sequence (5’-3’)** |
| --- | --- | --- | --- |
| mouse | *Gapdh-F* | AGGTCGGTGTGAACGGATTTG | |
|  | *Gapdh-R* | TGTAGACCATGTAGTTGAGGTCA | |
|  | *Col1a1-F* | GCTCCTCTTAGGGGCCACT | |
|  | *Col1a1-R* | CCACGTCTCACCATTGGGG | |
|  | *Timp1-F* | CTTGGTTCCCTGGCGTACTC | |
|  | *Timp1-R* | ACCTGATCCGTCCACAAACAG | |
|  | *Ctgf-F* | GGGCCTCTTCTGCGATTTC | |
|  | *Ctgf-R* | ATCCAGGCAAGTGCATTGGTA | |
|  | *Il-1β-F* | GCAACTGTTCCTGAACTCAACT | |
|  | *Il-1β-R* | ATCTTTTGGGGTCCGTCAACT | |
|  | *Tnf-α-F* | CCCTCACACTCAGATCATCTTCT | |
|  | *Tnf-α-R* | GCTACGACGTGGGCTACAG | |
|  | *Ap-1-F* | CCTTCACCCTGCCTCTTCTCA | |
|  | *Ap-1-R* | GGAACCAGACAGGTCCACATC | |
|  | *Cxcl1-F* | TCTCCGTTACTTGGGGAC | |
|  | *Cxcl1-R* | CCACACTCAAGAATGGTCGC | |
|  | *Ccl2-F* | TTAAAAACCTGGATCGGAACCAA | |
|  | *Ccl2-R* | GCATTAGCTTCAGATTTACGGGT | |
| human | *GAPDH-F* | GAGTCAACGGATTTGGTCGT | |
|  | *GAPDH-R* | GACAAGCTTCCCGTTCTCAG | |
|  | *TNF-α-F* | GGCAGTCAGATCATCTTCTCGAA | |
|  | *TNF-α-R* | GAAGGCCTAAGGTCCACTTGTGT | |
|  | *IL-1β-F* | ATGATGGCTTATTACAGTGGCAA | |
|  | *IL-1β-R* | GTCGGAGATTCGTAGCTGGA | |
|  | *IL-6-F* | AGACAGCCACTCACCTCTTCAG | |
|  | *IL-6-R* | TTCTGCCAGTGCCTCTTTGCTG | |
|  | *PCNA-F* | CCTGCTGGGATATTAGCTCCA | |
|  | *PCNA-R* | CAGCGGTAGGTGTCGAAGC | |
|  | *Ki67-F* | ACGCCTGGTTACTATCAAAAGG | |
|  | *Ki67-R* | CAGACCCATTTACTTGTGTTGGA | |

Table S3. Raw data of targeted LC-MS/MS lipidomics analysis.

|  | Ctrl | | | | | | Model | | | | | | NF | | | | | |
| --- | --- | --- | --- | --- | --- | --- | --- | --- | --- | --- | --- | --- | --- | --- | --- | --- | --- | --- |
| Compound | C1 | C2 | C3 | C4 | C5 | C6 | M1 | M2 | M3 | M4 | M5 | M6 | F1 | F2 | F3 | F4 | F5 | F6 |
| AA | 5.44E-02 | 3.93E-02 | 5.99E-02 | 3.00E-02 | 2.77E-02 | 3.78E-02 | 3.68E-02 | 4.27E-02 | 5.08E-02 | 8.10E-02 | 4.00E-02 | 4.39E-02 | 4.33E-02 | 1.47E-02 | 3.85E-02 | 2.71E-02 | 4.13E-02 | 2.73E-02 |
| 5-oxo-ETE-3 | 2.78E-05 | 6.17E-06 | 1.81E-05 | 9.88E-06 | 5.23E-05 | 1.33E-05 | 2.98E-05 | 2.53E-05 | 2.01E-05 | 2.20E-05 | 2.48E-05 | 3.54E-05 | 1.61E-05 | 9.30E-06 | 5.11E-05 | 8.80E-06 | 1.98E-05 | 3.17E-05 |
| 13-HODE | 2.33E-04 | 2.40E-04 | 1.21E-03 | 1.57E-04 | 1.07E-04 | 2.05E-04 | 0.000141 | 0.00291 | 0.001042 | 0.001226 | 0.000502 | 2.71E-04 | 1.47E-04 | 1.88E-04 | 0.000169 | 9.17E-04 | 2.81E-04 | 0.000256 |
| 9-HODE | 1.88E-04 | 1.84E-04 | 6.08E-04 | 1.10E-04 | 8.77E-05 | 1.39E-04 | 1.31E-04 | 1.09E-03 | 4.38E-04 | 7.73E-04 | 2.68E-04 | 2.16E-04 | 1.45E-04 | 1.27E-04 | 1.39E-04 | 2.30E-04 | 2.14E-04 | 0.000199 |
| 15-HETE | 5.59E-05 | 4.49E-05 | 8.90E-05 | 2.50E-05 | 2.32E-05 | 4.27E-05 | 3.30E-05 | 1.86E-04 | 8.70E-05 | 1.64E-04 | 3.52E-05 | 5.12E-05 | 3.58E-05 | 2.15E-05 | 3.39E-05 | 4.23E-05 | 4.26E-05 | 4.17E-05 |
| 12-HETE | 3.50E-04 | 2.22E-04 | 1.18E-03 | 1.85E-04 | 8.94E-05 | 1.72E-04 | 3.57E-04 | 3.73E-03 | 1.11E-03 | 1.39E-03 | 2.09E-04 | 2.72E-04 | 2.35E-04 | 5.23E-05 | 1.42E-04 | 3.32E-04 | 2.11E-04 | 0.000336 |
| 5-HETE | 2.45E-05 | 1.49E-05 | 3.22E-05 | 2.08E-05 | 1.32E-05 | 1.46E-05 | 1.01E-05 | 2.53E-05 | 2.36E-05 | 4.04E-05 | 1.92E-05 | 2.04E-05 | 2.20E-05 | 6.72E-06 | 3.63E-05 | 8.17E-06 | 1.35E-05 | 1.27E-05 |
| LTB4 | 2.49E-05 | 2.51E-05 | 2.58E-05 | 1.50E-05 | 1.95E-05 | 1.56E-05 | 8.65E-06 | 1.39E-05 | 1.57E-05 | 2.77E-05 | 2.80E-05 | 2.23E-05 | 1.85E-05 | 9.90E-06 | 2.15E-05 | 2.23E-05 | 1.43E-05 | 1.80E-05 |
| LTD4 | 1.93E-05 | 3.48E-05 | 2.45E-05 | 2.62E-05 | 2.29E-05 | 1.58E-05 | 1.65E-06 | 5.15E-06 | 1.92E-05 | 1.58E-05 | 1.52E-05 | 1.66E-05 | 2.55E-05 | 8.76E-06 | 1.98E-05 | 1.31E-05 | 4.31E-05 | 1.67E-05 |
| oxo-ETE | 2.78E-05 | 6.17E-06 | 1.81E-05 | 9.88E-06 | 5.23E-05 | 1.33E-05 | 2.98E-05 | 2.53E-05 | 2.01E-05 | 2.2E-05 | 2.48E-05 | 3.54E-05 | 1.61E-05 | 9.30E-06 | 5.11E-05 | 8.80E-06 | 1.98E-05 | 3.17E-05 |
| HODE | 4.20E-04 | 4.24E-04 | 1.82E-03 | 2.67E-04 | 1.95E-04 | 3.44E-04 | 2.72E-04 | 4.00E-03 | 1.48E-03 | 2.00E-03 | 7.71E-04 | 4.87E-04 | 2.92E-04 | 3.15E-04 | 3.08E-04 | 1.15E-03 | 4.95E-04 | 4.55E-04 |
| Leukotrienes | 4.42E-05 | 5.99E-05 | 5.03E-05 | 4.12E-05 | 4.24E-05 | 3.14E-05 | 1.03E-05 | 1.91E-05 | 3.49E-05 | 4.34E-05 | 4.32E-05 | 3.89E-05 | 4.40E-05 | 1.87E-05 | 4.13E-05 | 3.54E-05 | 5.74E-05 | 3.46E-05 |
| dhk PGF2a | 5.29E-06 | 5.67E-06 | 9.45E-06 | 5.83E-06 | 2.78E-06 | 8.46E-06 | 4.92E-06 | 1.04E-05 | 7.82E-06 | 1.45E-05 | 4.20E-06 | 3.97E-06 | 6.27E-06 | 3.46E-06 | 5.71E-06 | 6.67E-06 | 5.54E-06 | 6.86E-06 |
| dhk PGD2 | 1.19E-05 | 1.28E-05 | 1.36E-05 | 1.50E-05 | 1.63E-05 | 1.75E-05 | 2.54E-06 | 4.81E-06 | 1.23E-05 | 1.30E-05 | 1.08E-05 | 9.80E-06 | 9.36E-06 | 8.30E-06 | 2.12E-05 | 2.08E-05 | 1.08E-05 | 1.3E-05 |
| 6k-PGF1a | 3.82E-05 | 5.80E-05 | 9.05E-05 | 3.68E-05 | 2.05E-05 | 2.05E-05 | 7.10E-05 | 5.48E-04 | 3.15E-04 | 4.97E-04 | 1.38E-04 | 1.24E-04 | 4.87E-05 | 3.01E-05 | 1.81E-05 | 1.75E-04 | 8.32E-05 | 9.72E-05 |
| 11b PGF2a | 2.51E-06 | 1.22E-06 | 6.29E-06 | 6.96E-06 | 5.81E-06 | 3.96E-06 | 2.49E-05 | 4.95E-06 | 3.87E-06 | 5.52E-06 | 3E-06 | 2.6E-06 | 4.85E-06 | 1.75E-06 | 5.68E-06 | 8.05E-06 | 4.1E-07 | 6.18E-06 |
| PGF2a | 6.35E-06 | 1.84E-05 | 1.52E-05 | 3.77E-06 | 8.56E-06 | 1.07E-05 | 5.24E-06 | 1.86E-05 | 2.18E-05 | 4.46E-05 | 1.13E-05 | 1.61E-05 | 1.29E-05 | 4.59E-06 | 7.28E-06 | 6.27E-06 | 4.38E-06 | 1.03E-05 |
| PGF2b | 4.02E-06 | 9.86E-06 | 1.07E-05 | 2.73E-06 | 3.18E-06 | 6.63E-06 | 4.14E-06 | 1.52E-05 | 1.75E-05 | 3.04E-05 | 3.3E-06 | 8.27E-06 | 4.58E-06 | 1.83E-06 | 1.93E-06 | 3.23E-06 | 3.93E-06 | 4.34E-06 |
| PGE2 | 3.95E-06 | 9.68E-06 | 2.06E-05 | 4.28E-06 | 2.73E-06 | 2.43E-06 | 5.77E-06 | 7.75E-05 | 5.31E-05 | 9.91E-05 | 3.21E-06 | 8.92E-06 | 5E-06 | 2.76E-06 | 2.9E-06 | 1.29E-05 | 8.05E-06 | 8.09E-06 |
| PGD2 | 1.04E-05 | 1.49E-05 | 1.86E-05 | 7.35E-06 | 7.17E-06 | 6.36E-06 | 5.2E-05 | 6.33E-05 | 5.86E-05 | 1.13E-04 | 9.08E-06 | 1.73E-05 | 8.35E-06 | 3.82E-06 | 4.93E-06 | 2.12E-05 | 1.53E-05 | 1.3E-05 |
| PGB2 | 3.82E-05 | 6.40E-05 | 7.23E-05 | 4.99E-05 | 5.50E-05 | 6.52E-05 | 1.92E-05 | 3.79E-05 | 5.61E-05 | 6.98E-05 | 4.98E-05 | 4.13E-05 | 5.06E-05 | 2.34E-05 | 5.45E-05 | 5.85E-05 | 4.86E-05 | 4.26E-05 |
| 15-deoxy-PGJ2 | 1.89E-05 | 2.82E-05 | 2.63E-05 | 2.13E-05 | 2.06E-05 | 7.76E-06 | 3.50E-06 | 8.30E-06 | 1.41E-05 | 1.62E-05 | 3.14E-05 | 2.99E-05 | 4.11E-05 | 1.08E-05 | 4.10E-05 | 1.39E-05 | 5.80E-05 | 2.58E-05 |
| TXB2 | 6.34E-06 | 7.44E-06 | 8.14E-06 | 2.91E-06 | 1.91E-06 | 4.43E-06 | 1.82E-05 | 3.96E-05 | 1.54E-05 | 4.36E-05 | 4.08E-06 | 7.61E-06 | 4.40E-06 | 3.17E-06 | 3.33E-06 | 9.38E-06 | 3.71E-06 | 5.83E-06 |
| dhk-PG | 1.72E-05 | 1.85E-05 | 2.30E-05 | 2.08E-05 | 1.91E-05 | 2.60E-05 | 7.45E-06 | 1.52E-05 | 2.01E-05 | 2.75E-05 | 1.50E-05 | 1.38E-05 | 1.56E-05 | 1.18E-05 | 2.69E-05 | 2.75E-05 | 1.63E-05 | 1.99E-05 |
| PG | 1.38E-04 | 2.23E-04 | 2.81E-04 | 1.43E-04 | 1.43E-04 | 1.46E-04 | 1.89E-04 | 7.85E-04 | 5.60E-04 | 8.94E-04 | 2.60E-04 | 2.62E-04 | 1.96E-04 | 8.80E-05 | 1.55E-04 | 3.07E-04 | 2.39E-04 | 0.000227 |
| TXB | 7.04E-06 | 8.61E-06 | 9.45E-06 | 3.19E-06 | 2.61E-06 | 4.67E-06 | 1.86E-05 | 4.12E-05 | 1.65E-05 | 4.60E-05 | 4.62E-06 | 8.50E-06 | 4.86E-06 | 3.85E-06 | 4.09E-06 | 1.01E-05 | 3.96E-06 | 6.87E-06 |
| 12,13-diHOME | 3.45E-04 | 3.21E-04 | 6.57E-04 | 1.93E-04 | 2.11E-04 | 4.00E-04 | 9.97E-05 | 1.59E-04 | 3.79E-04 | 5.73E-04 | 4.04E-04 | 2.66E-04 | 3.11E-04 | 1.18E-04 | 2.88E-04 | 3.14E-04 | 2.77E-04 | 0.00024 |
| 9,10-diHOME | 1.77E-04 | 1.22E-04 | 3.35E-04 | 8.47E-05 | 1.18E-04 | 2.05E-04 | 7.19E-05 | 1.90E-04 | 1.51E-04 | 3.51E-04 | 1.52E-04 | 1.46E-04 | 1.21E-04 | 6.13E-05 | 1.54E-04 | 1.56E-04 | 1.46E-04 | 0.000122 |
| 12,13-EpOME | 6.70E-05 | 3.45E-05 | 9.15E-05 | 5.70E-05 | 5.57E-05 | 2.21E-05 | 1.88E-05 | 1.12E-04 | 9.28E-05 | 1.29E-04 | 4.18E-05 | 8.04E-05 | 6.12E-05 | 1.51E-05 | 6.05E-05 | 1.54E-05 | 8.38E-05 | 4.01E-05 |
| 9,10-EpOME | 4.63E-05 | 2.93E-05 | 7.10E-05 | 4.92E-05 | 3.40E-05 | 1.39E-05 | 1.01E-05 | 1.00E-04 | 7.05E-05 | 1.13E-04 | 2.48E-05 | 5.00E-05 | 4.42E-05 | 1.28E-05 | 5.02E-05 | 1.26E-05 | 5.34E-05 | 3.07E-05 |
| 11,12-EET | 1.33E-05 | 1.36E-05 | 5.46E-06 | 1.20E-06 | 1.1E-05 | 7.57E-06 | 3.15E-06 | 3.18E-05 | 4.87E-06 | 1.81E-05 | 1.66E-06 | 2.43E-05 | 7.87E-06 | 3.72E-06 | 1.36E-05 | 1.34E-06 | 6.2E-07 | 5.07E-06 |
| 11,12-DHET | 6.79E-05 | 3.31E-05 | 4.15E-05 | 1.96E-05 | 3.22E-05 | 3.86E-05 | 9.37E-06 | 9.89E-06 | 3.28E-05 | 3.92E-05 | 4.91E-05 | 3.28E-05 | 2.85E-05 | 1.41E-05 | 3.20E-05 | 2.84E-05 | 2.41E-05 | 2.38E-05 |
| 14,15-DHET | 1.94E-04 | 8.39E-05 | 7.69E-05 | 3.63E-05 | 6.66E-05 | 8.34E-05 | 1.57E-05 | 1.53E-05 | 7.55E-05 | 1.25E-04 | 8.48E-05 | 6.49E-05 | 7.41E-05 | 2.76E-05 | 4.95E-05 | 7.35E-05 | 4.54E-05 | 4.31E-05 |
| 8,9-DHET | 9.70E-05 | 3.79E-05 | 4.33E-05 | 2.68E-05 | 4.03E-05 | 5.43E-05 | 2.07E-05 | 1.52E-05 | 4.78E-05 | 7.21E-05 | 4.68E-05 | 2.24E-05 | 3.44E-05 | 1.49E-05 | 4.33E-05 | 4.38E-05 | 2.64E-05 | 2.65E-05 |
| 5,6-DHET | 9.30E-06 | 7.14E-06 | 4.58E-06 | 3.72E-06 | 1.01E-05 | 3.30E-06 | 1.83E-08 | 4.14E-06 | 9.34E-06 | 1.06E-05 | 9.29E-06 | 4.41E-06 | 1.06E-05 | 1.60E-06 | 4.43E-06 | 4.08E-06 | 4.18E-06 | 5.84E-06 |
| 18-HETE | 6.10E-06 | 1.16E-05 | 8.77E-06 | 3.75E-06 | 2.31E-06 | 8.03E-06 | 4.41E-06 | 2.93E-06 | 4.57E-06 | 1.03E-05 | 7.51E-06 | 4.79E-06 | 8.17E-06 | 6.58E-06 | 8.22E-06 | 8.30E-06 | 5.37E-06 | 7.05E-06 |
| 17-HETE | 4.70E-06 | 4.63E-06 | 7.30E-06 | 4.15E-06 | 4.21E-06 | 4.07E-06 | 6.46E-07 | 7.71E-07 | 9.37E-06 | 1.21E-05 | 5.29E-06 | 7.14E-06 | 5.62E-06 | 2.15E-06 | 4.72E-06 | 4.07E-06 | 5.22E-06 | 3.99E-06 |
| 16-HETE | 5.10E-06 | 4.24E-06 | 5.37E-06 | 2.24E-06 | 2.37E-06 | 6.20E-06 | 2.01E-06 | 2.55E-06 | 5.56E-06 | 6.04E-06 | 3.67E-06 | 4.65E-06 | 3.21E-06 | 3.16E-06 | 4.86E-06 | 5.15E-06 | 5.34E-06 | 1.32E-06 |
| diHOME | 5.22E-04 | 0.000444 | 9.92E-04 | 2.77E-04 | 3.29E-04 | 6.05E-04 | 0.000172 | 3.49E-04 | 0.00053 | 9.24E-04 | 5.56E-04 | 4.12E-04 | 4.32E-04 | 1.79E-04 | 4.42E-04 | 4.70E-04 | 4.23E-04 | 0.000363 |
| EpOME | 1.13E-04 | 6.37E-05 | 1.63E-04 | 1.06E-04 | 8.96E-05 | 3.60E-05 | 2.88E-05 | 2.12E-04 | 1.63E-04 | 2.42E-04 | 6.66E-05 | 1.30E-04 | 1.05E-04 | 2.78E-05 | 1.11E-04 | 2.80E-05 | 1.37E-04 | 7.08E-05 |
| EET | 1.33E-05 | 1.36E-05 | 5.46E-06 | 1.20E-06 | 1.10E-05 | 7.57E-06 | 3.15E-06 | 3.18E-05 | 4.87E-06 | 1.81E-05 | 1.66E-06 | 2.43E-05 | 7.87E-06 | 3.72E-06 | 1.36E-05 | 1.34E-06 | 6.20E-07 | 5.07E-06 |
| DHET | 3.68E-04 | 1.62E-04 | 1.66E-04 | 8.65E-05 | 1.49E-04 | 1.80E-04 | 4.58E-05 | 4.46E-05 | 1.66E-04 | 2.47E-04 | 1.90E-04 | 1.25E-04 | 1.48E-04 | 5.82E-05 | 1.29E-04 | 1.50E-04 | 1.00E-04 | 9.92E-05 |
| HETE | 4.46E-04 | 3.02E-04 | 1.32E-03 | 2.41E-04 | 1.35E-04 | 2.47E-04 | 4.07E-04 | 3.95E-03 | 1.24E-03 | 1.63E-03 | 2.80E-04 | 3.60E-04 | 3.09E-04 | 9.24E-05 | 2.30E-04 | 4.00E-04 | 2.83E-04 | 0.000402 |

### **Supplementary References**

[1] Colyn L, Bárcena-Varela M, Álvarez-Sola G, et al. Dual Targeting of G9a and DNA Methyltransferase-1 for the Treatment of Experimental Cholangiocarcinoma. Hepatology (Baltimore, Md). 2021;73:2380-2396.

[2] Hasan S, Ghani N, Zhao X, et al. Dietary pyruvate targets cytosolic phospholipase A2 to mitigate inflammation and obesity in mice. Protein & cell. 2024;15:661-685.

[3] Hirano Y, Gao Y-G, Stephenson DJ, et al. Structural basis of phosphatidylcholine recognition by the C2-domain of cytosolic phospholipase A2α. eLife. 2019;8:e44760.

[4] Das A, Jana G, Sing S, et al. Insights into the interaction and inhibitory action of palmatine on lysozyme fibrillogenesis: Spectroscopic and computational studies. International journal of biological macromolecules. 2024;268:131703.
